# Cell-based therapies for experimental chronic kidney disease: a systematic review and meta-analysis

**DOI:** 10.1242/dmm.017699

**Published:** 2015-01-29

**Authors:** Diana A. Papazova, Nynke R. Oosterhuis, Hendrik Gremmels, Arianne van Koppen, Jaap A. Joles, Marianne C. Verhaar

**Affiliations:** Department of Nephrology and Hypertension, University Medical Centre Utrecht, 3508 GA Utrecht, The Netherlands

**Keywords:** Cell-based therapy, Chronic kidney disease, Meta-analysis

## Abstract

Cell-based therapy is a promising strategy for treating chronic kidney disease (CKD) and is currently the focus of preclinical studies. We performed a systematic review and meta-analysis to evaluate the efficacy of cell-based therapy in preclinical (animal) studies of CKD, and determined factors affecting cell-based therapy efficacy in order to guide future clinical trials. In total, 71 articles met the inclusion criteria. Standardised mean differences (SMD) and 95% confidence intervals (CI) were calculated for outcome parameters including plasma urea, plasma creatinine, urinary protein, blood pressure, glomerular filtration rate, glomerulosclerosis and interstitial fibrosis. Sub-analysis for each outcome measure was performed for model-related factors (species, gender, model and timing of therapy) and cell-related factors (cell type, condition and origin, administration route and regime of therapy). Overall, meta-analysis showed that cell-based therapy reduced the development and progression of CKD. This was most prominent for urinary protein (SMD, 1.34; 95% CI, 1.00–1.68) and urea (1.09; 0.66–1.51), both *P*<0.001. Changes in plasma urea were associated with changes in both glomerulosclerosis and interstitial fibrosis. Sub-analysis showed that cell type (bone-marrow-derived progenitors and mesenchymal stromal cells being most effective) and administration route (intravenous or renal artery injection) were significant predictors of therapeutic efficacy. The timing of therapy in relation to clinical manifestation of disease, and cell origin and dose, were not associated with efficacy. Our meta-analysis confirms that cell-based therapies improve impaired renal function and morphology in preclinical models of CKD. Our analyses can be used to optimise experimental interventions and thus support both improved preclinical research and development of cell-based therapeutic interventions in a clinical setting.

## INTRODUCTION

Worldwide, the number of individuals with chronic kidney disease (CKD) is rising, mainly owing to a dramatic increase in atherosclerosis and type-2 diabetes (Khwaja et al., 2007). CKD is a progressive condition causing significant morbidity and mortality. The ensuing end-stage renal disease and associated increase in cardiovascular risk represent a significant socio-economic burden. Slowing CKD progression is therefore a major health priority.

Cell-based therapy has proven to be a promising clinical approach for several pathological conditions and might represent a novel therapeutic strategy to slow the progression of kidney disease (Tögel and Westenfelder, 2012). Preclinical studies have demonstrated beneficial effects of various (stem) cell populations and cell-derived factors – secreted growth factors, microvesicles and exosomes – in acute kidney injury models, suggesting a renal regenerative effect of cell-based therapies. Importantly, these preclinical observations have already translated into pioneering clinical trials. Recently, a phase I clinical trial showed that administration of allogeneic mesenchymal stem cells (MSCs) to open-heart surgery patients at high risk of acute renal failure was feasible and safe (Tögel and Westenfelder, 2012). Furthermore, MSCs are being used in several clinical trials in kidney transplant recipients with the aim of increased immunosuppression and improved regeneration (Reinders et al., 2013a; Tan et al., 2012).

CKD is characterised by reduced renal regenerative capacity. Several studies suggest beneficial regenerative effects of cell-based therapies in animal models of CKD. However, it is unclear which cell types or cell products improve renal function and morphology most effectively in experimental CKD. The design of preclinical studies is very diverse, varying in terms of models of CKD, timing of interventions, cell type or cell product, number of cells, administration route and read-out of kidney function and morphology, which makes translation to the clinic difficult. A meta-analysis and systematic review of existing animal studies will facilitate the design of future clinical studies. Moreover, the information obtained can be used to optimise existing experimental animal models and interventions and thus to improve preclinical research in the future. We have performed a systematic review and meta-analysis in order to evaluate the effect of cell-based therapy on kidney function and morphology outcome parameters, and we have analysed cell- and model-related aspects. To identify potential bona fide markers of target organ injury in the setting of cell-based therapy, we performed a correlation analysis between functional data (blood pressure and blood and urinary markers) and morphological data [glomerulosclerosis (GS) and tubular interstitial fibrosis (IF)].

## RESULTS

### Study selection and characteristics

**Fig. 1.**
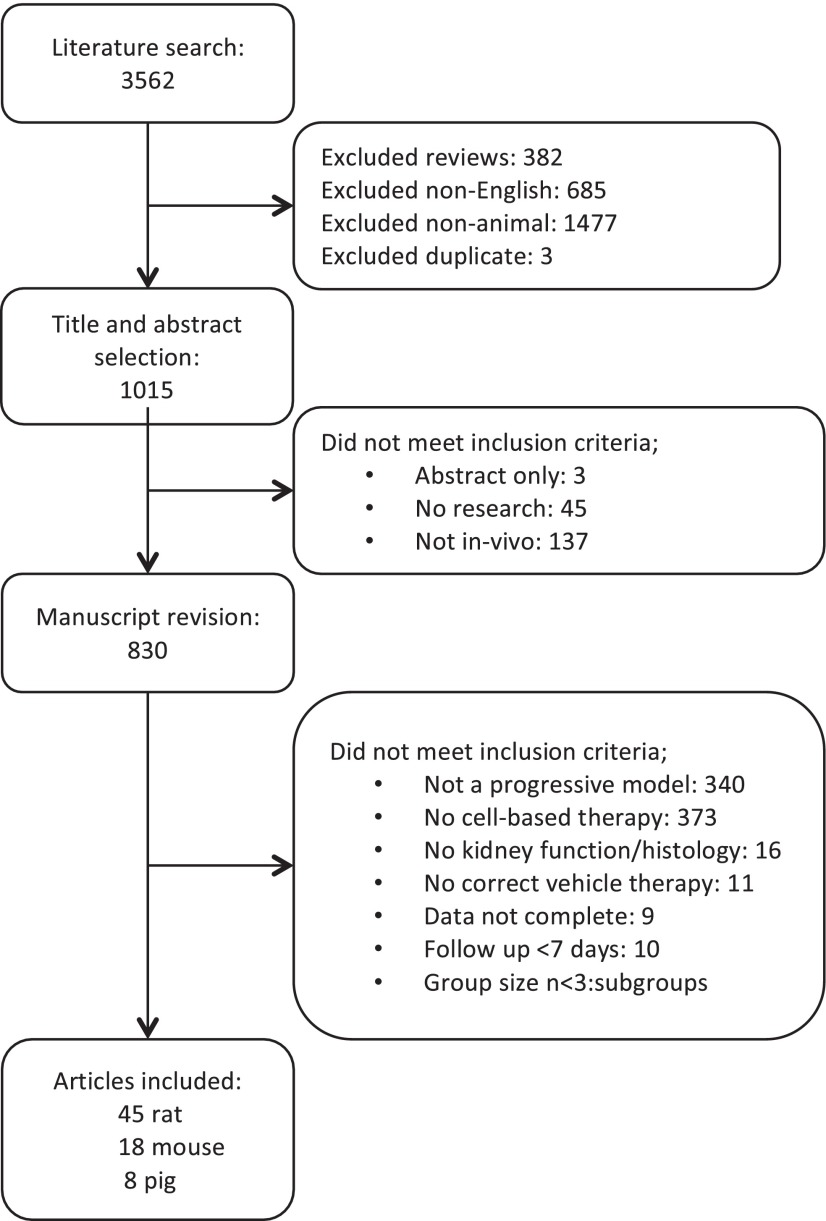
**Flow chart of study selection.** Articles were selected according to inclusion and exclusion criteria defined in the Materials and Methods section.

Our electronic search strategy delivered 1015 articles from PubMed database, 944 of which were excluded because inclusion criteria were not met. Data were extracted from 71 articles (Fig. 1). A large variation in study characteristics was observed (supplementary material Table S1). Cell types and products were pooled to facilitate analysis (supplementary material Tables S2, S3). Most studies used MSCs (58%) to evaluate their therapeutic efficacy on CKD. Only three studies evaluated both preventive as well as rescue cell-based interventions, whereas 38% studied only preventive and 58% only rescue interventions. Most studies used single administration (68%), 23% used multiple administrations (two to eight times) and 9% of the studies investigated both. Of all five cell-delivery routes (renal artery, intra-arterial non-renal, intravenous, parenchymal or subcapsular, and intraperitoneal), intravenous cell administration was used in the majority of studies (68%). A total of 1813 animals were used to investigate the effect of cell-based therapies on CKD – 442 mice, 1244 rats and 127 pigs, representing 1056 male and 585 female animals. Ten studies did not report the gender of the animals. In rats and mice, CKD was induced using 19 different models that we first pooled into the following groups: subtotal nephrectomy (SNX), diabetic nephropathy (DN), ischemia-reperfusion injury, genetic non-diabetes and hypertension (supplementary material Table S3). Studies in pigs all used renal artery stenosis to induce kidney injury.

**TRANSLATIONAL IMPACT**

**Clinical issue**

Despite elucidation of major factors involved in the progression of chronic kidney disease (CKD) and development of better treatments, the number of individuals with end-stage kidney disease (ESKD) that require renal replacement therapy is steadily increasing. Preclinical studies have suggested beneficial effects of cell-based therapy but this has not yet been translated to the clinic. A systematic review and meta-analysis of animal studies of cell-based therapy in CKD is useful in order to facilitate the translation of this knowledge into therapies for individuals with CKD or even ESKD.

**Results**

In the 71 articles that met the inclusion criteria for this study, 1813 animals were used: 442 mice, 1244 rats and 127 pigs. Cell-based therapy improved all functional and histological outcome parameters and reduced development and progression of CKD. Mesenchymal stromal cells, bone marrow progenitor cells and endothelial progenitor cells were the most effective cell-based therapies, and intravenous and intrarenal artery were the most effective delivery routes. Single and multiple administrations were equally effective and no dose response in delivered cell number was found. Changes in plasma urea correlated significantly with changes in two morphological parameters, glomerulosclerosis and interstitial fibrosis.

**Implications and future directions**

This systematic review and meta-analysis confirms that cell-based therapies can effectively improve impaired renal function and morphology in animal models of CKD. These results can be used to optimize experimental models and interventions and thus improve preclinical research and support development of cell-based therapeutic interventions in a clinical setting.

### Meta-analysis of outcome measures

The efficacy of cell-based therapy in treating CKD was assessed using the following functional parameters: plasma creatinine, plasma urea, glomerular filtration rate (GFR), blood pressure (BP) and urinary protein. We analysed 51 studies that measured plasma creatinine (mice, 10; pigs, 5; rats, 36) and 27 studies that measured plasma urea (mice, 8; rats, 19). The GFR analysis contained studies that reported inulin (rats, 6) or creatinine clearance (15 studies: mice, 2; rats, 13) and 8 studies with multi-detector computed tomography (MDCT, all in pigs). BP analysis included 10 studies in rats and 8 in pigs, and urinary protein analysis included 46 studies (mice, 10; pigs, 3; rats, 33). We also analysed the histological parameters GS [28 studies (mice, 8; pigs, 3; rats, 17)] and IF [27 studies (mice, 2; pigs, 8; rats, 17)].

**Fig. 2.**
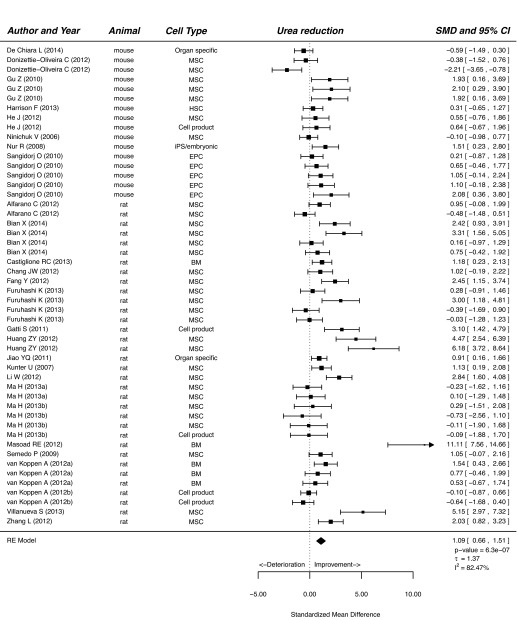
**The effect of cell-based treatment of CKD on plasma urea.** Forest plot; the right side shows improvement by cell-based therapy. Data are presented as SMDs and 95% Cl. Only the first author of each paper is shown. iPS, induced pluripotent stem cell; RE, random effects.

**Fig. 3.**
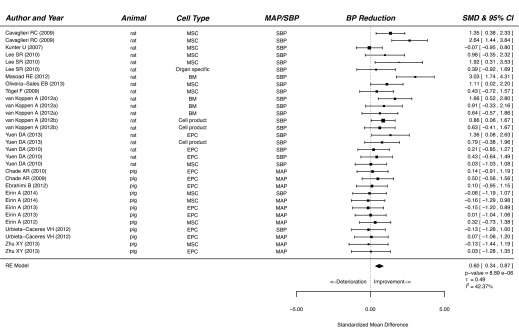
**The effect of cell-based treatment of CKD on blood pressure.** Forest plot; the right side shows improvement by cell-based therapy. Data are presented as SMDs and 95% Cl. Only the first author of each paper is shown. MAP, mean arterial pressure; SBP, systolic blood pressure; RE, random effects.

**Fig. 4.**
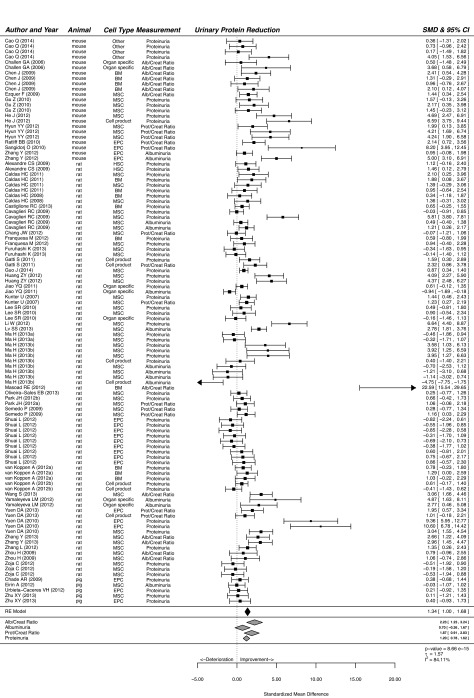
**The effect of cell-based treatment of CKD on urinary protein.** Forest plot; the right side shows improvement by cell-based therapy. Data are presented as SMDs and 95% Cl. Only the first author of each paper is shown. RE, random effects.

**Fig. 5.**
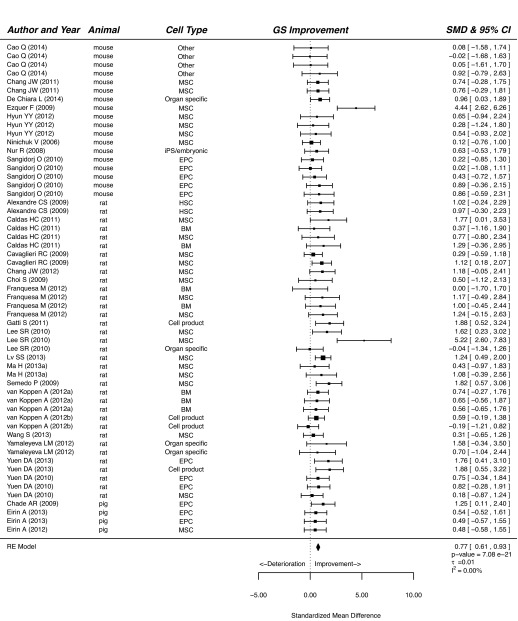
**The effect of cell-based treatment of CKD on glomerulosclerosis.** Forest plot; the right side shows improvement by cell-based therapy. Data are presented as SMDs and 95% Cl. Only the first author of each paper is shown. iPS, induced pluripotent stem cell; RE, random effects.

**Fig. 6.**
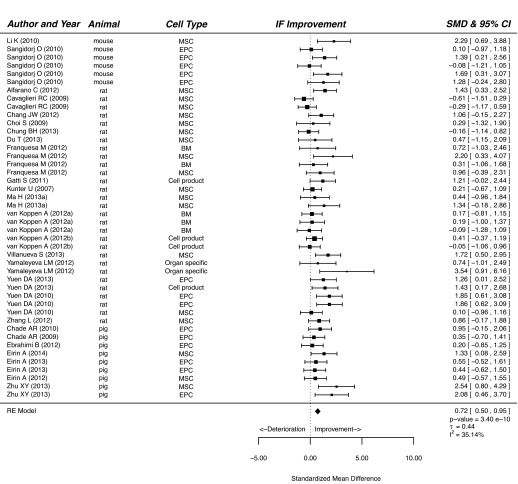
**The effect of cell-based treatment of CKD on interstitial fibrosis.** Forest plot; the right side shows improvement by cell-based therapy. Data are presented as SMDs and 95% Cl. Only the first author of each paper is shown. RE, random effects.

Our meta-analysis showed that treatment of CKD with cells or cell products significantly improved functional and histological parameters. Results for the effect of cell-based therapy on plasma creatinine are summarised in supplementary material Fig. S1. Plasma creatinine decreased after cell-based therapy compared with that of vehicle-treated or control animals (SMD, 0.98; 95% CI, 0.73, 1.24; *P*<0.001). Similarly, cell-based therapy reduced plasma urea in experimental CKD (SMD, 1.09; 95% CI, 0.66, 1.51; *P*<0.001; Fig. 2). Cell-based therapy increased GFR (SMD, 1.05; 95% CI, 0.67, 1.43; *P*<0.001; supplementary material Fig. S2) and decreased BP (SMD, 0.60; 95% CI, 0.34, 0.87; *P*<0.001; Fig. 3) compared with that of control animals. The outcome measure most amenable to improvement by cell-based therapy was urinary protein (SMD, 1.34; 95% CI, 1.00, 1.68; *P*<0.001; Fig. 4). Reductions in GS (SMD, 0.77; 95% CI, 0.61, 0.93; *P*<0.001; Fig. 5) and IF (SMD, 0.72; 95% CI 0.50, 0.95; *P*<0.001; Fig. 6) were observed after cell-based therapy. Heterogeneity in effect size between studies was high (>72%) for all functional outcome parameters except for BP, where it was moderate (42%). No heterogeneity was detected between studies that measured GS. Heterogeneity between studies that measured IF was moderate (35%).

### Correlations between functional measurements and renal tissue injury

**Fig. 7.**
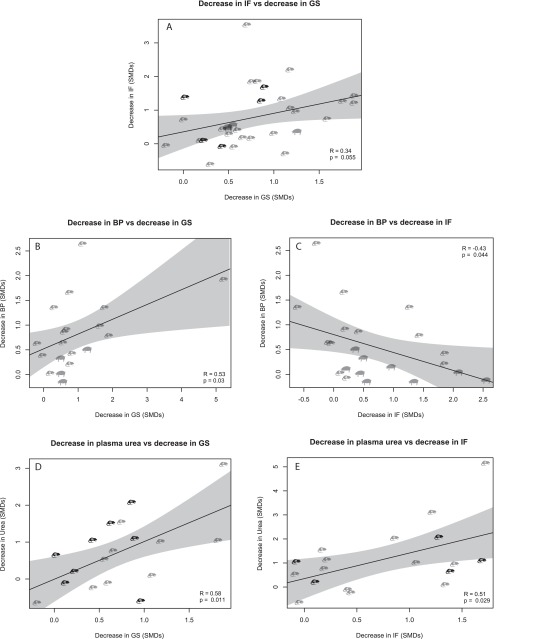
**Correlations between renal functional parameters and tissue injury parameters.** (A) Correlation between decrease in interstitial fibrosis (IF) versus decrease in glomerulosclerosis (GS). (B) Correlation between decrease in blood pressure (BP) versus decrease in GS. (C) Correlation between decrease in BP versus decrease in IF. (D) Correlation between decrease in plasma urea versus decrease in GS. (E) Correlation between decrease in plasma urea versus decrease in GS. M, mouse; r, rat.

No significant correlation was found between reductions in GS and IF (*R*=0.34, *P*=0.055, Fig. 7A). Therefore, we hypothesised that correlations between functional outcome measures and structural outcome measures would be different for GS and IF. Indeed, reduction in BP correlated positively with reduction in GS (*R*=0.53, *P*=0.030, Fig. 7B), but negatively with reduction in IF (*R*=−0.43, *P*=0.044, Fig. 7C). Urinary protein, the marker that was most strongly affected by cell-based therapy (Fig. 4) did not correlate with either reduction in GS (*R*=0.12, *P*=0.41, supplementary material Fig. S3A) or reduction in IF (*R*=0.28, *P*=0.13, supplementary material Fig. S3B). Reduction in plasma urea correlated positively with reduction in both GS (*R*=0.58, *P*=0.011, Fig. 7D) and IF (*R*=0.51, *P*=0.029, Fig. 7E). Reduction in plasma creatinine correlated with GS (*R*=0.48, *P*=0.003, supplementary material Fig. S3C), but not with IF (*R*=0.32, *P*=0.069, supplementary material Fig. S3D) and increased GFR did not correlate with GS (*R*=0.35, *P*=0.086, supplementary material Fig. S3E), but correlated strongly with IF (*R*=0.69, *P*<0.001, supplementary material Fig. S3F).

### Subgroup-analysis and meta-regression

#### Cell-based-treatment-related factors

##### Cell type

Meta-regression showed that for most outcome measures, differences in the administered cell type did not explain variations in treatment effect. The most evidence currently supports MSC treatment, as MSC treatment consistently improved all functional and histological parameters except GFR (supplementary material Fig. S4A–G), the largest decrease being observed for urinary protein (SMD, 1.49; 95% CI, 0.97, 2.02; *P*<0.001; supplementary material Fig. S4E). Bone marrow cells (BM) had beneficial effects on urea, BP, urinary protein and GS (supplementary material Fig. S4B,D–F). Only GFR showed a significant difference between cell types, with endothelial progenitor cells (EPCs) causing significantly more improvement in GFR than other cell types (*P*<0.001, supplementary material Fig. S4C).

##### Delivery route

When delivered intravenously, cell treatment improved all functional and histological parameters, with the greatest increase observed on GFR (SMD, 1.51; 95% CI, 0.85, 2.18; *P*<0.001; supplementary material Fig. S4C). Cell administration directly via the renal artery was also effective on all functional parameters except GFR (supplementary material Fig. S4C). All other cell types or products [hematopoietic stem cells (HSCs), embryonic and organ specific] and delivery routes (intraperitoneal, intra-arterial non-renal and parenchyma or subcapsular) were applied too infrequently to be reliably interpreted.

##### Regime

We observed no difference between single and multiple cell administration regimes, except for GFR, where multiple administrations showed no effect (supplementary material Fig. S4C). Both regimes reduced the development and progression of CKD as shown by reduced creatinine, urea, BP and urinary protein and less GS and IF (supplementary material Fig. S4A,B,D–G). For none of the outcome parameters was there a significant relationship between administered cell number and effect size, even though administered cell number between studies differed by more than four orders of magnitude (data not shown).

##### Cell origin and condition

Both xenogeneic (human cells administered to rodents) and allogeneic (animals receiving cells from the same species) transplantation of cells were effective at improving outcome parameters, except for GFR, where xenotransplantation failed to improve this parameter (supplementary material Fig. S4A–G). The majority of studies used cells from healthy donors and ‘healthy cells’ consistently improved functional and histological outcome parameters.

##### Model-related factors (species, gender, model and timing of therapy)

All studies that used rats to study the efficacy of cell-based treatment in CKD showed improvement in all functional and histological parameters (supplementary material Fig. S5A–G). Cell therapy did not influence urea and GFR in mice (supplementary material Fig. S5B,C), and did not influence creatinine, BP and urinary protein in pigs (supplementary material Fig. S5A,D,E). Gender did not affect the outcome of cell therapy except in the case of GFR, which was not improved in males (supplementary material Fig. S5C). Heterogeneity in design was too substantial to identify differential treatment effects between different disease models. The most commonly used model, SNX, showed consistent improvements in all functional and histological parameters, with the biggest increase for GFR (SMD, 1.78; 95% CI, 1.20, 2.36; *P*<0.001; supplementary material Fig. S5C). Diabetes models generally seem to show greater improvements than other models (except for GFR), although the power is too limited to achieve statistical significance (supplementary material Fig. S5A–G). Both preventive and rescue cell-based treatments improved development and progression of CKD, shown by all outcome measures (supplementary material Fig. S5). The timing of therapy in relation to clinical manifestation of disease (prevention or rescue treatment) was not associated with efficacy.

### Quality assessment

The results of methodological quality assessment are shown in supplementary material Fig. S6. All included studies were assessed using 13 characteristics. Of the assessed characteristics, 70% were scored positive. Eight articles were of high quality (>80% positive characteristics) and two articles were of low quality (<50% positive characteristics). Numbers of animals, CKD model, follow-up, dosage, administration route, timing of intervention and outcome measures were all adequately reported. Animal characteristics were often only partly described (52 out of 71 articles), with strain, age, body weight and/or gender sometimes not being mentioned. Randomisation of animals was only reported in 40% of all studies. Scoring and analysis of histology parameters was only performed blindly by the researchers in a third of all studies.

### Publication bias and sensitivity analysis

Publication bias was assessed for all outcome measures. Visual assessment of funnel plots showed no publication bias for plasma urea, BP, GS and IF (supplementary material Fig. S7A,E,F). Small negative studies appeared to be underrepresented for plasma creatinine, urinary protein excretion and GFR (supplementary material Fig. S7B–D) and Egger’s test showed asymmetry for these three outcome measures (*P*<0.001). The ‘trim and fill’ method showed that there are seven hypothetical studies missing for creatinine, three for urinary protein and one for GFR (imputed in supplementary material Fig. S8). A re-run of the meta-analysis for those three outcome measures, including computed studies, showed very similar effects to the original results.

## DISCUSSION

Our systematic review and meta-analysis showed that cell-based therapy reduced the development and progression of experimental CKD, as measured by several commonly and clinically used measures of renal function (creatinine, urea, GFR, BP and urinary protein) and for common experimentally used measures of renal damage (GS and IF). This finding proved to be consistent despite considerable differences between studies in the selection and preparation of cells, administration route and choice of disease model and model species.

Before considering cell- and model-related factors that might influence the efficacy of cell-based therapy, we analysed whether correlation analysis could be of use in identifying potential bona fide markers of target organ injury. The rationale for this approach was that for ethical reasons only animal studies allow systematic quantitative analysis of target organ injury. Thus, correlation between functional and structural data in animal studies might affect our perspective of commonly used functional markers in the setting of cell-based therapy. Upfront, we need to acknowledge that, owing to limited availability of raw data, this analysis was performed with SMDs and was not weighted for study precision. Nevertheless, the outcome was illuminative.

First, we analysed whether reductions in GS and IF were correlated. Surprisingly, reductions in GS and IF did not correlate significantly, suggesting that, at least partly, correlations with functional data would be different for GS and IF. Indeed, we observed a positive correlation between reductions in GS and BP and a negative correlation between IF and BP. The positive correlation between GS and BP is in line with the results found in the SNX model in rats (Griffin et al., 2004), suggesting that BP-dependent mechanisms are important for the development of GS. However, the negative correlation between change in BP and change in IF was unexpected. Importantly, changes in urinary protein do not correlate significantly with changes in either GS or IF. This is quite different from the results of multiple studies with blockade of the renin angiotensin system (Sarafidis and Ruilope, 2014), suggesting that a different mechanism might be involved in cell-based treatment. In the correlation analysis, we found that, generally, changes in plasma markers and direct measurements of GFR correlate differently with changes in GS than with changes in IF. The notable exception is that a change in plasma urea shows consistent correlations with changes in both GS and IF. That changes in urea predict changes in IF is well known from biopsy studies (Bohle et al., 1996); however, for GS this is less well documented. All in all, changes in BP, plasma urea and plasma creatinine appear to be good predictors of changes in GS, whereas, for IF, plasma urea and measured GFR are the most important. The lack of significant correlations with urinary protein for either GS or IF suggests that changes in this outcome predict functional rather than structural changes. However, it should be noted that we could not study temporal relations in our meta-analysis.

We performed subgroup analyses and meta-regression to investigate predefined factors that we hypothesised would modify the efficacy of cell-based treatment in CKD – cell-related factors (cell type, regime, condition, origin and delivery route) and model-related factors (species, gender, model and timing of intervention). Our meta-analysis most strongly supports the use of MSCs as therapy for CKD, although studies using BM and EPC seem to achieve similar results. MSCs are currently under investigation for a wide range of clinical applications, as they possess anti-inflammatory, anti-fibrotic and proangiogenic properties (Lalu et al., 2012). Clinical trials with MSCs have been initiated for acute kidney injury and transplantation (Cantaluppi et al., 2013), but application of MSCs in the setting of CKD has not yet taken place. There remain important issues in MSC therapy that need to be addressed before the translation to clinical studies can be made. The majority of the studies in our meta-analysis were performed with MSCs from donors free of kidney disease, with only one study testing cells that originated from uremic donors (van Koppen et al., 2012a). Uremia has been suggested to induce functional incompetence in BM-MSCs (Klinkhammer et al., 2014; Kramann et al., 2011; Noh et al., 2012) but neither subcutaneous-adipose-tissue-derived MSCs nor bone-marrow-derived MSCs obtained from individuals with renal disease showed persistent dysfunction in *in vitro* assays after expansion in culture (Reinders et al., 2013b; Roemeling-van Rhijn et al., 2012). Similarly, *in vivo* studies show no persistent dysfunction in pro-angiogenic effects of MSCs obtained from diseased individuals (Gremmels et al., 2014). Whether the uremic environment is detrimental for cell-based therapy requires further investigation. Importantly, the low antigenicity and immunomodulatory properties of MSCs allow allogeneic transplantation, which could lead to an ‘off-the-shelf’ therapy. In general, the use of human cells in ~25% of all the experimental animal studies resulted in favourable results, even though the recipients were usually immune competent.

Cell products are also attractive candidates for off-the-shelf therapy. However, only six studies using cell products were available for our meta-analysis, prohibiting definitive conclusions. One might speculate that, in the chronic situation, multiple administrations of cells or cell products would confer benefits over single administration because paracrine actions might decrease over time. Similar considerations could be held regarding the number of administered cells. However, our meta-analysis showed no dose dependency, either in the number of cells or cell product administrations or in cell or cell product dose. Lack of dose dependency is a common finding in cell-based therapy (van der Spoel et al., 2011), possibly suggesting that cell-based therapy acts primarily by switching on endogenous repair rather than as a persistent source of exogenous cells or growth factors. Indeed, multiple clinical and experimental studies fail to find substantial numbers of exogenous cells in the kidney after their administration (Choi et al., 2010).

Systemic intravenous delivery (through the tail vein in most rodent studies) was the route that was most supported by evidence, despite the fact that the majority of administered cells appear to be trapped in the lungs (Fischer et al., 2009). This also suggests that even relatively few cells passing the pulmonary circulation are sufficient to switch on endogenous repair. This delivery route is feasible for patients, because injecting intravenously is relatively easy and minimally invasive. In patients, intravenous infusions of MSCs were well tolerated and no treatment-related serious adverse events are reported (Reinders et al., 2013a). Direct intrarenal delivery was applied in 17 articles in our meta-analysis – five using subcapsular or parenchymal administration and 12 using delivery by injection in the renal artery. These studies generally show improved outcome measures, although findings were less consistent than with intravenous administration. In conjunction with their more invasive character, this makes these approaches less attractive, although theoretically combination with other common endovascular treatments of the renal artery (denervation or stenting) is attractive. Intraperitoneal delivery was only used in three studies, none of which showed a significantly improved outcome. Based on these limited findings, intraperitoneal delivery of cell-based therapy in CKD does not appear to be useful.

Our meta-analysis suggests differences in the efficacy of cell-based therapy between species in urinary protein and BP, but not in other outcome measures. Partly, such differences might be due to methodological limitations; for instance, BP measurements were practically absent in the mouse studies included in our meta-analysis. However, importantly, cell-based therapy improved GS and IF in all three species. Thus, for structural changes, all three species appear to be useful, although in pigs protective effects on GS were limited, albeit significant.

We did not observe consistent effects of gender on the outcome of cell therapy, except for improvements in GFR, which only occurred in studies using female animals. Mechanisms underlying gender-specific differences in outcome measures of cell therapy are obscure, and cannot be clarified by a meta-analysis. Nevertheless, the possibility that this is also the case in humans should be taken into account when designing cell-therapy studies in patients, and gender balance should be considered. Furthermore, differences in the functional efficacy of cell-based therapy in CKD appeared to be model dependent, perhaps reflecting the different pathogenesis of CKD when initiated by subtotal nephrectomy versus, for instance, toxic injury. However, for structural efficacy, most models showed improvement of all outcome variables, perhaps reflecting the common pathway to end-stage kidney disease. Such differences might also be relevant when designing cell-therapy studies in specific patient populations.

Preventive and rescue interventions are both effective. However, the staging of CKD in animals is not as clearly defined as in humans, and therapy was never instituted at a late stage that clinically would be equivalent to pre-dialysis. Moreover, preventive therapy in animal models is often initiated directly after (or even before) renal ablation or administration of toxins, well before any GS or IF can be expected. Thus, the relevance of this finding to the clinical situation is, perhaps, limited. Nevertheless, it could be that in clinical studies very tight categorisation of CKD stage is not required to find significant effects of cell-based therapy.

Clearly, both human and animal studies should be performed according to the highest standards. Although randomisation and blinding for inclusion is often not feasible in the setting of an experimental study, blinding for analysis of both functional and structural parameters is readily achievable. Moreover, there is no excuse for not reporting all relevant animal characteristics as well as the number of drop-outs due to technical failures or premature (non-scheduled) death or sacrifice. Omitting such data is very common in animal studies, including many studies in our meta-analysis, and has been reported in other meta-analyses (van der Spoel et al., 2011; Wever et al., 2012). It is crucial that authors report these details, as they will likely influence outcome parameters.

Our meta-analysis confirms that cell-based therapies improve impaired renal function and structure in preclinical models of CKD. Animal studies are often regarded with scepticism because of large variation in study design and outcome measures. However, our meta-analysis demonstrates that perhaps because of this very variation they can be perceived as a rich source of useful information that could be helpful in designing clinical trials in the (near) future.

## MATERIALS AND METHODS

### Literature search

We conducted a systematic review and meta-analysis of studies that investigated the effects of cell- and cell-based therapies on kidney function and structure in animal models of CKD. The PubMed database was searched for published articles up to 21 January 2014 using the following terms: ‘kidney damage’ [Tiab] OR ‘kidney injury’ [Tiab] OR ‘kidney disease’ [Tiab] OR ‘renal injury’ [Tiab] OR ‘renal failure’ [Tiab] OR ‘kidney failure’ [Tiab] OR ‘nephropathy’ [Tiab] OR ‘renal disease’ [Tiab] OR ‘renal function’ [Tiab] OR ‘renovascular’ [Tiab] AND ‘stem cells’ [Tiab] OR ‘cell therapy’ [Tiab] OR ‘progenitor cells’ [Tiab] OR ‘bone marrow’ [Tiab] OR EPC [Tiab] OR ‘endothelial progenitor cells’ [Tiab] OR ‘MSC’ [Tiab] OR ‘mesenchymal’ [Tiab] OR ‘conditioned medium’ [Tiab] OR ‘microvesicles’ [Tiab] OR ‘exosomes’ [Tiab] OR ‘microparticles’ [Tiab]. Search results were filtered by PubMed filters to exclude reviews and non-English articles, and a custom-made filter (Hooijmans et al., 2010) was used to select for studies containing laboratory animals. Articles were selected by reading the title and abstract; when these were not informative enough, the complete article was screened by two independent researchers (D.A.P. and N.R.O.). Articles were discussed with the other authors before inclusion or exclusion.

### Inclusion criteria

The criteria for inclusion were as follows: (1) animal models of CKD were used to study the effects of cell-based therapy on kidney function and structure; (2) the therapy contained or consisted of cells or cell-derived products (conditioned medium or exosomes or microvesicles) and (3) the article was an original paper presenting unique data.

### Exclusion criteria

The criteria for exclusion were as follows: (1) animal models of acute renal failure showing spontaneous recovery of kidney injury under the untreated condition. These models included ischemia-reperfusion injury, anti-Thy1 and various toxic models (cisplatin, glycerol, gentamicin, mercuric chloride, folic acid, carbon tetrachloride and lipopolysaccharide; (2) follow-up was less than seven days after disease induction; (3) group size smaller than *n*=3; (4) incomplete data (no untreated or vehicle-treated diseased control group present; missing data at last measurement point before or at termination; data from figures or tables with unclear captions; unknown group size and/or standard deviations or errors of mean); (5) no full-text article available.

### Data extraction

Study characteristics extracted from selected articles included species, strain, gender, injury model, administered cell type or cell-derived product, number of cells, administration route, time-point of administration, follow-up duration. Data analysis for kidney function included plasma creatinine, plasma urea, GFR, BP and urinary protein [proteinuria, albuminuria, urine protein:creatinine (prot:creat) and urine albumin:creatinine (alb:creat) ratio]. Data analysis for renal structure included GS and IF. For longitudinal measurements, data from the last measurement (function) or at termination (morphology) were used. When one control group was used for comparison with multiple treatment groups, control sample size was divided by the number of treatment groups to equalise the weight of each group in our analysis (Vesterinen et al., 2014). Plasma and serum urea, blood urea nitrogen (BUN) and plasma and serum creatinine levels shown as mg/dl were converted to mmol/l (mmol/l= BUN mg/dl×0.357 or mmol/l=plasma urea mg/dl×0.1665) and μmol/l (plasma/serum creatinine μmol/l=mg/dl×88.4), respectively. Both plasma creatinine and creatinine clearance were analyzed when reported in one article. BP measurements (systolic blood pressure and mean arterial pressure) of conscious animals were included to avoid the effects of anaesthesia. Standard error of the mean (s.e.m.) was converted to standard deviation (s.d., s.d.=√n×s.e.m.). When necessary, GetData Graph Digitizer (version 2.25) was used to extract values from graphs. Data were extracted by two independent researchers (D.A.P. and N.R.O.). Missing data were requested from authors.

### Data analysis

Data were analyzed with R software (version 3.1.0; R Foundation for Statistical Computing, Vienna, Austria) using the metafor package (Viechtbauer, 2010). In order to correct for the different units and scales arising from the abovementioned considerations, all data are presented as standardised mean differences (SMDs). SMDs and accompanying variance were calculated for the following outcome parameters: plasma urea, plasma creatinine, urinary protein, GFR, BP, GS and IF. Random- and mixed-effects models were fitted using restricted maximum likelihood estimation (REML). The estimated average effect (μ), heterogeneity in effects (τ^2^) and the estimated percentage of variability attributable to heterogeneity (I^2^) are given. Heterogeneity was considered to be low, moderate or high at 25, 50 and 75%, respectively (Higgins et al., 2003). In order to study the relationship between improvements in renal morphology (GS and IF) with different outcome measures related to renal function, we calculated pair-wise correlations between the SMDs. Pearson product-moment correlation coefficients are supplied, with bands indicating 95% confidence intervals.

We studied the influence of several moderators on treatment effect using meta-regression. All models only employ a single moderator variable, as study designs were too diverse to get full models without empty cells in case of multiple factor models. Moderators were grouped in cell treatment-related moderators (cell type, administration regime, condition of cells, origin of cells, administration route) and model-related (animal model species, gender, model, timing of intervention) factors. These factors were pooled in classes for sub-analysis because of the wide variety in cell types and animal models (supplementary material Tables S2, S3). Sub-analysis for each outcome variable was performed for all cell treatment- and model-related factors. In rodents, hypertension-induced kidney injury was used in one study; owing to this low number, data are only shown in figures, and not taken into account when interpreting results. Porcine studies were only included for species sub-analysis because in all porcine studies the same model (renal artery stenosis) was applied. All cell-based therapies were categorised as preventive or rescue. A treatment was defined as preventive when cell-based therapy was applied before clinical manifestation of disease (for induced models, between day 0 and day 6 after induction of kidney disease; for knockout models, before clinical manifestation of disease). Therapies were categorised as rescue when therapy was started after clinical manifestation of disease.

### Quality assessment

Methodological quality of the included studies was assessed by a scoring system adapted from Wever et al. (Wever et al., 2012). Publication bias was assessed by visually evaluating asymmetry in funnel plots for each outcome parameter. In case of visual asymmetry, Egger’s test was used (Sterne and Egger, 2005). When Egger’s test indicated asymmetry, the ‘trim and fill’ method (Duval and Tweedie, 2000) was used to correct for this.

## Supplementary Material

Supplementary Material
