# Supplementary material for: Cell-based therapies for experimental chronic kidney disease: a systematic review and meta-analysis
Source: Dis Model Mech. 2015 Jan 29;8(3):281–93. doi: 10.1242/dmm.017699 (PMC4348565; doi:10.1242/dmm.017699)
Supplement: Supplementary Material [file supp_8_3_281__index.html]

Cell-based therapies for experimental chronic kidney disease: a systematic review and meta-analysis — Supplementary Material 

# Cell-based therapies for experimental chronic kidney disease: a systematic review and meta-analysis

## DMM017699 Supplementary Material

**Files in this Data Supplement:**

- **Supplementary Material**
