# Supplementary material for: Cell-based therapies for experimental chronic kidney disease: a systematic review and meta-analysis
Source: Dis Model Mech. 2015 Jan 29;8(3):281–93. doi: 10.1242/dmm.017699 (PMC4348565; doi:10.1242/dmm.017699)

Supplementary material

Title: Cell-based therapies for experimental chronic kidney disease: a systematic review and meta-analysis

Authors: Diana A. Papazova*, Nynke R. Oosterhuis*, Hendrik Gremmels*, Arianne van Koppen, Jaap A. Joles, Marianne C. Verhaar
*Equal contribution

Affiliation: Department of Nephrology & Hypertension, University Medical Centre Utrecht, Netherlands

Table S1. Study characteristics (A- animal strain; G-gender; NV – number of animals in vehicle group; NC – number animals in cell-based treated group). Other abbreviations are listed in the legends of Tables S1 and S2.

											Days after induction	
**Author, year**	**A**	**Strain**	**G**	**NV**	**NC**	**Model**	**Cell type**	**# cells**	**Route**	**Timing**	**Start therapy**	**End study**
(Alexandre et al., 2009)	rat	Fischer 344	M	8	8	5/6 Nx	Lin^- BM	$2*10^6$	Intravenous	Rescue	15	60
(Alexandre et al., 2009)	rat	Fischer 344	M		8	5/6 Nx	Lin^- BM	$2*10^6$	Intravenous	Rescue	15, 30, 45	60
(Alexandre et al., 2009)	rat	Fischer 344	M	12	7	5/6 Nx	Lin^- BM	$2*10^6$	Intravenous	Rescue	15	120
(Alfarano et al., 2012)	rat	Lewis	F	8	8	Unx + IRI + CsA	MSC melatonin pretreated	$3*10^6$	Parenchymal	Rescue	7	28
(Alfarano et al., 2012)	rat	Lewis	F	8	8	Unx + IRI + CsA	MSC melatonin pretreated	$3*10^6$	Parenchymal	Rescue	14	28
(Bian et al., 2014)	rat	Sprague-Dawley	M	6	6	5/6 Nx	MSC	$10*10^6$	Intravenous	Rescue	28	56
(Bian et al., 2014)	rat	Sprague-Dawley	M	6	6	5/6 Nx	MSC	$10*10^6$	Intravenous	Rescue	56	84
(Bian et al., 2014)	rat	Sprague-Dawley	M	6	6	5/6 Nx	MSC	$10*10^6$	Intravenous	Rescue	84	112
(Bian et al., 2014)	rat	Sprague-Dawley	M	6	6	5/6 Nx	MSC	$10*10^6$	Intravenous	Rescue	112	140
(Burst et al., 2013)	rat	Lewis	F	7	7	Unx + IRI	Lin^- $CD90^+$HSC	$1*10^6$	Intra-arterial	Prevention	0	7
(Caldas et al., 2011)	rat	Wistar	F	5	5	2/3 Nx	MSC	$2,5*10^6$	Scaffold kidney	Prevention	0	90
(Caldas et al., 2011)	rat	Wistar	F		5	2/3 Nx	BMMNC	$5*10^6$	Scaffold kidney	Prevention	0	90
(Caldas et al., 2011)	rat	Wistar	F	5	5	5/6 Nx	MSC	$2,5*10^6$	Scaffold kidney	Prevention	0	90
(Caldas et al., 2011)	rat	Wistar	F		5	5/6 Nx	BMMNC	$5*10^6$	Scaffold kidney	Prevention	0	90
(Caldas et al., 2008)	rat	Wistar	M	5	5	5/6 Nx	BMMNC	$1,5*10^6$	Parenchymal	Prevention	0	119
(Caldas et al., 2008)	rat	Wistar	M		5	5/6 Nx	MSC	$1,5*10^6$	Parenchymal	Prevention	0	119
(Cantaluppi et al., 2012)	rat	Wistar	M	6	6	Unx + IRI	EPC MV	30ug	Intravenous	Prevention	0	180
(Cao et al., 2013)	mouse	Balb/c	M	7	7	Adriamycin	BM-M0	$1*10^6$	Intravenous	Prevention	5	28

(Cao et al., 2013)	mouse	Balb/c	M		7	Adriamycin	BM-M2	$1*10^6$	Intravenous	Prevention	5	28
(Cao et al., 2013)	mouse	Balb/c	M		7	Adriamycin	SP-M0	$1*10^6$	Intravenous	Prevention	5	28
(Cao et al., 2013)	mouse	Balb/c	M		7	Adriamycin	SP-M2	$1*10^6$	Intravenous	Prevention	5	28
(Castiglione et al., 2013)	rat	Wistar	M	10	10	DN	BMMNC	$20*10^6$	Intravenous	Rescue	28	112
(Cavaglieri et al., 2009)	rat	Wistar	M	10	10	5/6 Nx	MSC	2*105	Subcapsular	Prevention	0	15
(Cavaglieri et al., 2009)	rat	Wistar	M	10	10	5/6 Nx	MSC	$2*10^5$	Subcapsular	Prevention	0	30
(Chade et al., 2010)	pig	domestic	?	7	7	RAS	EPC	$10*10^6$	Intrarenal artery	Rescue	42	70
(Chade et al., 2009)	pig	domestic	?	7	7	RAS	EPC	$10*10^6$	Intrarenal artery	Rescue	42	70
(Challen et al., 2006)	mouse	BALB/c	?	3	3	Adriamycin	MP	$3*10^5$	Parenchymal/ intrarenal artery	Prevention	0	7
(Challen et al., 2006)	mouse	BALB/c	?	3	3	Adriamycin	SP	$3*10^5$	Parenchymal/ intrarenal artery	Prevention	0	7
(Chang et al., 2012)	rat	Sprague-Dawley	M	6	6	5/6 Nx	chMSC	$1*10^6$	Intravenous	Prevention	0, 14, 28, 42, 56, 70, 84, 98	112
(Chang et al., 2011)	mouse	NZB/W F1	F	8	8	Lupus nephritis	hU-MSC	$1*10^6$	Intravenous	Prevention	61	244
(Chang et al., 2011)	mouse	NZB/W F1	F	8	7	Lupus nephritis	hU-MSC	$1*10^6$	Intravenous	Rescue	183	244
(Chen et al., 2009)	mouse	Db/Db	M	5	6	DN	BMMNC db/m	$1*10^6$	Intravenous	Rescue	112, 122, 132, 142	161
(Chen et al., 2009)	mouse	Db/Db	M		6	DN	BMMNC db/db	$1*10^6$	Intravenous	Rescue	112, 122, 132, 142	161
(Chen et al., 2009)	mouse	Db/Db	M	4	5	DN	BMMNC db/db ex vivo Ebselen	$1*10^6$	Intravenous	Rescue	112, 122, 132, 142	161
(Chen et al., 2009)	mouse	Db/Db	M		5	DN	BMMNC db/db in vivo Ebselen	$1*10^6$	Intravenous	Rescue	112, 122, 132, 142	161
(Choi et al., 2009)	rat	Sprague-Dawley	F	3	3	5/6 Nx	MSC	$1*10^6$	Intravenous	Prevention	1	60
(Chung et al., 2013)	rat	Sprague-Dawley	M	8	8	CsA	hADMSC	$3*10^6$	Intravenous	Prevention	0, 7, 14, 21	28
(De Chiara et al., 2014)	mouse	129sv/C57	F	10	10	Unx + IRI	GTC	$2.5*10^5$	Intravenous	Prevention	0	42
(Donizetti-Oliveira et al., 2012)	mouse	C57BL/6J	F	6	6	IRI	ADSC	$2*10^5$	Intraperitoneal	Prevention	0	42
(Donizetti-Oliveira et al., 2012)	mouse	C57BL/6J	F	6	6	IRI	ADSC	$2*10^5$	Intraperitoneal	Rescue	42	70
(Du et al., 2013)	rat	Sprague-Dawley	M	3	3	IRI	hWJ-MSC	$2*10^6$	Intravenous	Prevention	2	42
(Ebrahimi et al., 2012)	pig	Domestic	F	7	7	RAS	EPC	$1*10^6$	Intrarenal artery	Rescue	42	70
(Eirin et al., 2014)	pig	Domestic	F	6	6	RAS	ADMSC	$10*10^6$	Intrarenal artery	Rescue	42	77
(Eirin et al., 2013)	pig	Domestic	?	7	7	RAS	EOC	$10*10^6$	Intrarenal artery	Rescue	42	70
(Eirin et al., 2013)	pig	Domestic	?	7	7	RAS + PTRA	EOC	$10*10^6$	Intrarenal artery	Rescue	42	70
(Eirin et al., 2012)	pig	Domestic	F	7	7	RAS	ADMSC	$10*10^6$	Intrarenal artery	Rescue	42	70
(Ezquer et al., 2009)	mouse	C57BL/6J	M	8	8	DN	MSC	$0,5*10^6$	Intravenous	Rescue	30, 51	119
(Fang et al., 2012)	rat	Sprague-Dawley	M	8	8	DN	ADMSC	$10*10^6$	Intravenous	Rescue	28	84
(Franquesa et al., 2012)	rat	Lewis	M	4	4	CAN	BM	$0,5*10^6$	Intravenous	Rescue	77	84
(Franquesa et	rat	Lewis	M	4	7	CAN	MSC	$10*10^6$	Intravenous	Rescue	77	84

al., 2012)												
(Franquesa et al., 2012)	rat	Lewis	M	7	5	CAN	BM	0,5*10^6	Intravenous	Rescue	77	168
(Franquesa et al., 2012)	rat	Lewis	M	7	7	CAN	MSC	10*10^6	Intravenous	Rescue	77	168
(Furuhashi et al., 2013)	rat	WKY/NCrj	F	7	13	anti-GBM GN	MSC	2*10^6	Intravenous	Prevention	0, 1, 2, 3, 4, 5	7
(Furuhashi et al., 2013)	rat	WKY/NCrj	F		7	anti-GBM GN	HASC	2*10^6	Intravenous	Prevention	0, 1, 2, 3, 4, 5	7
(Furuhashi et al., 2013)	rat	WKY/NCrj	F	7	7	anti-GBM GN	HASC	2*10^6	Intravenous	Rescue	0, 1, 2, 3, 4, 5	14
(Furuhashi et al., 2013)	rat	WKY/NCrj	F		8	anti-GBM GN	LASC	2*10^6	Intravenous	Rescue	0, 1, 2, 3, 4, 5	14
(Gatti et al., 2011)	rat	Sprague-Dawley	M	6	6	UNX + IRI	hMSC MV	30ug	Intravenous	Prevention	0	182
(Gu et al., 2010)	mouse	MRL/lpr	F	6	8	Lupus nephritis	hU-MSC	1*10^6	Intravenous	Rescue	126	203
(Gu et al., 2010)	mouse	MRL/lpr	F		8	Lupus nephritis	hU-MSC	1*10^6	Intravenous	Rescue	126, 133, 140	203
(Gu et al., 2010)	mouse	MRL/lpr	F		8	Lupus nephritis	MSC	1*10^6	Intravenous	Rescue	126	203
(Guo et al., 2014)	rat	Sprague-Dawley	M	30	30	Adriamycin	MSC	2*10^6	Intravenous	Rescue	14, 21	70
(Harrison et al., 2013)	mouse	Ctns(-/-)	M	9	8	Cystinosis	HSPC	1*10^6	Intravenous	Rescue	77	365
(He et al., 2012)	mouse	C57BL6/J	?	6	10	5/6 Nx	MSC	1*10^6	Intravenous	Prevention	2	9
(He et al., 2012)	mouse	C57BL6/J	?		10	5/6 Nx	MSC MV	30ug	Intravenous	Prevention	2, 3, 5	9
(Huang et al., 2012)	rat	?	?	10	10	NSN	MSC	0,3*10^5	Intrarenal artery	Prevention	6	21
(Huang et al., 2012)	rat	?	?		10	NSN	GDNF-MSC	0,3*10^5	Intrarenal artery	Prevention	6	21
(Hyun et al., 2012)	mouse	HIGA	F	7	5	IgAN	ADSC preonset disease	5*10^6	Intravenous	Rescue	168, 182, 196, 210, 224, 238	252
(Hyun et al., 2012)	mouse	HIGA	F		6	IgAN	ADSC postonset disease	5*10^6	Intravenous	Rescue	168, 182, 196, 210, 224, 238	252
(Hyun et al., 2012)	mouse	HIGA	F		8	IgAN	hADSC	5*10^6	Intravenous	Rescue	168, 182, 196, 210, 224, 238	252
(Jiao et al., 2011)	rat	Sprague-Dawley	F	15	15	Adriamycin	MMC	5-7*10^6	Intravenous	Rescue	56	112
(Kunter et al., 2007)	rat	Lewis	M	10	10	Unx + aThy	MSC	2*10^6	Intrarenal artery	Prevention	2	60
(Lee et al., 2010)	rat	Sprague-Dawley	F	8	16	5/6 Nx	MSC	3*10^6	Intravenous	Prevention	1	56
(Lee et al., 2010)	rat	Sprague-Dawley	F		8	5/6 Nx	MSC	3*10^6	Intravenous	Prevention	1, 7, 14, 21, 28	56
(Lee et al., 2010)	rat	Sprague-Dawley	F		16	5/6 Nx	MC	3*10^6	Intravenous	Prevention	1	56
(Li et al., 2012)	rat	Wistar	F	10	10	CAAN	MSC	20*10^6	Intravenous	Rescue	28	84
(Li et al., 2010)	mouse	C57BL/6	?	5	5	IRI	hADMSC	5*10^5	Intravenous	Prevention	0	183
(Lv et al., 2013)	rat	Wistar	F	16	16	DN	MSC	2*10^6	Intravenous	Rescue	56	112
(Ma, H. et al., 2013)	rat	Sprague-Dawley	M	6	6	Adriamycin	hU-MSC	2*10^6	Intravenous	Prevention	1	84
(Ma, H. et al., 2013)	rat	Sprague-Dawley	M		6	Adriamycin	hU-MSC	2*10^6	Intravenous	Prevention	1, 8, 15, 22	84
(Ma, Hualin et al., 2013)	rat	Sprague-Dawley	M	6	6	Adriamycin	hU-MSC	2*10^6	Intravenous	Prevention	1	28
(Ma, Hualin et al., 2013)	rat	Sprague-Dawley	M		6	Adriamycin	hU-MSC	2*10^6	Intravenous	Prevention	1, 8, 15, 22	28
(Ma, Hualin et al., 2013)	rat	Sprague-Dawley	M		6	Adriamycin	hU-MSC	2*10^6	Intraperitoneal	Prevention	1, 8, 15, 22	28
(Ma, Hualin et al., 2013)	rat	Sprague-Dawley	M		6	Adriamycin	supernatant hU-MSC	2*10^6	Intravenous	Prevention	1, 8, 15, 22	28
(Masoad et al., 2012)	rat	?	M	10	10	DN	hUB-MNC	150*10^6	Intravenous	?	?	8

(Ninichuk et al., 2006)	mouse	COL4A3 KO	?	10	10	Alport syndrome	MSC	$1*10^6$	Intravenous	Rescue	42, 49, 56, 63	66
(Nur et al., 2008)	mouse	TC KO	M	6	6	HSV	DFAT	$5*10^6$	Intravenous	Prevention	0	21
(Oliveira-Sales et al., 2013)	rat	Wistar	M	8	7	2K-1C	MSC	$2.5*10^5$	Intravenous	Rescue	21, 35	42
(Park, J. H. et al., 2012)	rat	Sprague-Dawley	M	7	7	DN	hUB-SC	$1*10^6$	Intravenous	Rescue	28	70
(Park, Jong Hee et al., 2012)	rat	Sprague-Dawley	M	7	7	DN	hUB-MSC	$0,5*10^6$	Intravenous	Prevention	2	28
(Ratliff et al., 2010)	mouse	Balb/c	?		6	Adriamycin	EPC	$5*10^5$	Intravenous	Prevention	0	21
(Sangidorj et al., 2010)	mouse	C57BL/6	M	4	20	5/6 Nx	EPC	$1*10^6$	Intravenous	Prevention	1, 7	28
(Sangidorj et al., 2010)	mouse	C57BL/6	M	4	16	5/6 Nx	EPC	$1*10^6$	Intravenous	Prevention	1, 7	56
(Sangidorj et al., 2010)	mouse	C57BL/6	M	4	12	5/6 Nx	EPC	$1*10^6$	Intravenous	Prevention	1, 7	84
(Sangidorj et al., 2010)	mouse	C57BL/6	M	4	8	5/6 Nx	EPC	$1*10^6$	Intravenous	Prevention	1, 7	112
(Sangidorj et al., 2010)	mouse	C57BL/6	M	4	4	5/6 Nx	EPC	$1*10^6$	Intravenous	Prevention	1, 7	140
(Semedo et al., 2010)	mouse	C57BL/6	F	5	5	IRI	BM-MNC	$1*10^6$	Intraperitoneal	Prevention	0	42
(Semedo et al., 2009)	rat	Wistar	F	7	7	5/6 Nx	MSC	$2*10^5$	Intravenous	Rescue	14	84
(Semedo et al., 2009)	rat	Wistar	F	7	7	5/6 Nx	MSC	$2*10^5$	Intravenous	Rescue	14, 28, 42	56
(Shuai et al., 2012)	rat	Sprague-Dawley	F	8	8	5/6 Nx	EPC	$1*10^5$	Intravenous	Rescue	7	28
(Shuai et al., 2012)	rat	Sprague-Dawley	F		8	5/6 Nx	empty plasmid-EPC	$1*10^5$	Intravenous	Rescue	7	28
(Shuai et al., 2012)	rat	Sprague-Dawley	F		8	5/6 Nx	TERT-EPC	$1*10^5$	Intravenous	Rescue	7	28
(Shuai et al., 2012)	rat	Sprague-Dawley	F	8	8	5/6 Nx	EPC	$1*10^5$	Intravenous	Rescue	7	56
(Shuai et al., 2012)	rat	Sprague-Dawley	F		8	5/6 Nx	empty plasmid-EPC	$1*10^5$	Intravenous	Rescue	7	56
(Shuai et al., 2012)	rat	Sprague-Dawley	F		8	5/6 Nx	TERT-EPC	$1*10^5$	Intravenous	Rescue	7	56
(Shuai et al., 2012)	rat	Sprague-Dawley	F	8	8	5/6 Nx	EPC	$1*10^5$	Intravenous	Rescue	7	84
(Shuai et al., 2012)	rat	Sprague-Dawley	F		8	5/6 Nx	empty plasmid-EPC	$1*10^5$	Intravenous	Rescue	7	84
(Shuai et al., 2012)	rat	Sprague-Dawley	F		8	5/6 Nx	TERT-EPC	$1*10^5$	Intravenous	Rescue	7	84
(Togel et al., 2009)	rat	Sprague-Dawley	M	6	6	IRI	MSC	$1,5*10^6$	Intra-arterial	Prevention	0	90
(Togel et al., 2009)	rat	Sprague-Dawley	M	6	6	IRI	MSC	$0,5*10^6$	Intra-arterial	Prevention	0	90
(Togel et al., 2009)	rat	Sprague-Dawley	M		6	IRI	MSC	$2*10^6$	Intra-arterial	Prevention	0	90
(Togel et al., 2009)	rat	Sprague-Dawley	M		6	IRI	MSC	$5*10^6$	Intra-arterial	Prevention	0	90
(Togel et al., 2009)	rat	F334	M	6	6	IRI	MSC	$0,5*10^6$	Intra-arterial	Prevention	0	90
(Togel et al., 2009)	rat	F334	M		6	IRI	MSC	$2*10^6$	Intra-arterial	Prevention	0	90
(Togel et al., 2009)	rat	F334	M		6	IRI	MSC	$5*10^6$	Intra-arterial	Prevention	0	90
(Urbieta-Caceres et al., 2012)	pig	Domestic	?	6	6	RAS	EPC	$10*10^6$	Intrarenal artery	Rescue	42	70
(van Koppen et al., 2012b)	rat	Lewis	M	8	8	5/6 Nx	BM	$50*10^6$	Intrarenal artery	Rescue	42	84
(van Koppen et al., 2012b)	rat	Lewis	M	9	7	5/6 Nx	BM	$50*10^6$	Intrarenal artery	Rescue	42	140
(van Koppen et al., 2012b)	rat	Lewis	M		7	5/6 Nx	CKD BM	$50*10^6$	Intrarenal artery	Rescue	42	140

(van Koppen et al., 2012a)	rat	Lewis	M	13	13	5/6 Nx	hMSC CM	50 μg/250 μl	Intravenous	Rescue	42, 43, 44, 45	84
(van Koppen et al., 2012a)	rat	Lewis	M	7	8	5/6 Nx	hMSC Exo	7μg/250 μl	Intravenous	Rescue	42, 43, 44, 45	84
(Villanueva et al., 2013)	rat	Sprague-Dawley	M	7	7	5/6 Nx	hADMSC	$0,5*10^6$	Intravenous	Prevention	0	35
(Villanueva et al., 2011)	rat	Sprague-Dawley	M	7	7	5/6 Nx	MSC	$0,5*10^6$	Intravenous	Rescue	35	42
(Wang et al., 2013)	rat	Sprague-Dawley	M	8	9	DN	MSC	$2*10^6$	Intrarenal artery	Rescue	28	56
(Yamaleyeva et al., 2012)	rat	RH-Foxn1rnu	M	2	4	IRI + gentamicin	hPKC	$5*10^6$	Parenchymal	Rescue	126	210
(Yamaleyeva et al., 2012)	rat	RH-Foxn1rnu	M	2	4	IRI + gentamicin	F^{+}hPKC	$5*10^6$	Parenchymal	Rescue	126	210
(Yuen et al., 2013)	rat	F344	M	9	8	5/6 Nx	EOC	$1*10^6$	Intravenous	Rescue	28	56
(Yuen et al., 2013)	rat	F344	M		9	5/6 Nx	EOC CM	0.5 ml	Intravenous	Rescue	28, 30, 32, 35, 37, 39	56
(Yuen et al., 2010)	rat	F334	M	15	11	5/6 Nx	BM CMC	$1*10^6$	Intra-arterial	Rescue	28	56
(Yuen et al., 2010)	rat	F334	M		11	5/6 Nx	BM CMC	$1*10^6$	Intravenous	Rescue	28	56
(Yuen et al., 2010)	rat	F334	M		11	5/6 Nx	SC	$1*10^6$	Intravenous	Rescue	28	56
(Zhang, Y. et al., 2013)	rat	Sprague-Dawley	M	10	10	DN	MSC	$1*10^6$	Intravenous	Rescue	28	56
(Zhang, Y. et al., 2013)	rat	Sprague-Dawley	M		10	DN	microbubble MSC	$1*10^6$	Intravenous	Rescue	28	56
(Zhang, L. et al., 2013)	rat	Sprague-Dawley	M	8	8	DN	hASC	$5*10^6$	Intravenous	Rescue	84, 112, 140, 168, 196	224
(Zhang et al., 2012)	mouse	Db/Db	M	12	12	DN	EOC db/m	$0,5*10^6$	Intravenous	Rescue	56	84
(Zhang et al., 2012)	mouse	Db/Db	M		12	DN	EOC db/db	$0,5*10^6$	Intravenous	Rescue	56	84
(Zhou et al., 2009)	rat	Sprague-Dawley	M	4	4	DN	MSC	$2*10^6$	Cardiac infusion	Rescue	28	84
(Zhou et al., 2009)	rat	Sprague-Dawley	M		4	DN	MSC + CsA	$2*10^6$	Cardiac infusion	Rescue	28	84
(Zhu et al., 2013)	pig	Domestic	F	7	6	RAS	MSC	$10*10^6$	Intrarenal artery	Rescue	42	70
(Zhu et al., 2013)	pig	Domestic	F		6	RAS	EPC	$10*10^6$	Intrarenal artery	Rescue	42	70
(Zoja et al., 2012)	rat	Lewis	M	4	4	Adriamycin	MSC	$2*10^6$	Intravenous	Prevention	1, 2, 3, 5, 7	9
(Zoja et al., 2012)	rat	Lewis	M	4	4	Adriamycin	MSC	$2*10^6$	Intravenous	Prevention	1, 2, 3, 5, 7, 14	16
(Zoja et al., 2012)	rat	Lewis	M	4	4	Adriamycin	MSC	$2*10^6$	Intravenous	Prevention	1, 2, 3, 5, 7, 14, 21	30

References

Alexandre, C. S., Volpini, R. A., Shimizu, M. H., Sanches, T. R., Semedo, P., Jura, V. L. D., CÃ¢mara, N. O., Seguro, A. C. and Andrade, L. (2009). Lineage-negative bone marrow cells protect against chronic renal failure. *Stem Cells* **27**, 682.

Alfarano, C., Roubeix, C., Chaaya, R., Ceccaldi, C., Calise, D., Mias, C., Cussac, D., Bascands, J. L. and Parini, A. (2012). Intraparenchymal Injection of Bone Marrow Mesenchymal Stem Cells Reduces Kidney Fibrosis After Ischemia-Reperfusion in Cyclosporine-Immunosuppressed Rats. *Cell Transplantation* **21**, 2009.

Bian, X., Zhang, B., Guo, W., Liu, N., Bai, Y., Miao, J., Zhao, G., Liu, B., Wang, S., Ma, L. et al. (2014). Effects of Mesenchymal Stem Cells Transplanted at Different Time Points in a Rat Remnant Kidney Model. *American Journal of Nephrology* **39**, 75-84.

Burst, V., Putsch, F., Kubacki, T., Volker, L. A., Bartram, M. P., Muller, R. U., Gillis, M., Kurschat, C. E., Grundmann, F., Muller-Ehmsen, J. et al. (2013). Survival and distribution of injected haematopoietic stem cells in acute kidney injury. *Nephrology, dialysis, transplantation : official publication of the European Dialysis and Transplant Association - European Renal Association* **28**, 1131-1139.

Caldas, H. C., Fernandes, I. M. M., Kawasaki-Oyama, R. S., Baptista, M. A. S. F., Plepis, A. M. G., Martins, V. A., Coimbra, T. M., Goloni-Bertollo, E. M., Braile, D. M. and Abbud-Filho, M. (2011). Effect of stem cells seeded onto biomaterial on the progression of experimental chronic kidney disease. *Proceedings of the Society for Experimental Biology and Medicine*, ebm.2011.011024.
Caldas, H. C., Fernandes, I. M. M., Gerbi, F., Souza, A. C., Baptista, M. A. S. F., Ramalho, H. J., Kawasaki-Oyama, R. S., Goloni-Bertollo, E. M., Pavarino-Bertelli, E. C., Braile, D. M. et al. (2008). Effect of Whole Bone Marrow Cell Infusion in the Progression of Experimental Chronic Renal Failure. *Transplantation Proceedings* **40**, 853.

Cantaluppi, V., Gatti, S., Medica, D., Figliolini, F., Bruno, S., Deregibus, M. C., Sordi, A., Biancone, L., Tetta, C. and Camussi, G. (2012). Microvesicles derived from endothelial progenitor cells protect the kidney from ischemia-reperfusion injury by microRNA-dependent reprogramming of resident renal cells. *Kidney Int* **82**, 412.

Cao, Q., Wang, Y., Zheng, D., Sun, Y., Wang, C., Wang, X. M., Lee, V. W. S., Zheng, G., Tan, T. K., Wang, Y. M. et al. (2013). Failed renoprotection by alternatively activated bone marrow macrophages is due to a proliferation-dependent phenotype switch in vivo. *Kidney International*.

Castiglione, R. C., Maron-Gutierrez, T., Barbosa, C. M. L., Ornellas, F. M., Barreira, A. L., Dibarros, C. B. A., Vasconcelos-Dos-Santos, A., Paredes, B. D., Pascarelli, B. M., Diaz, B. L. et al. (2013). Bone marrow-derived mononuclear cells promote improvement in glomerular function in rats with early diabetic nephropathy. *Cellular Physiology and Biochemistry* **32**, 699-718.

Cavaglieri, R. C., Martini, D., Sogayar, M. C. and Noronha, I. L. (2009). Mesenchymal Stem Cells Delivered at the Subcapsule of the Kidney Ameliorate Renal Disease in the Rat Remnant Kidney Model. *Transplantation Proceedings* **41**, 947.

Chade, A. R., Zhu, X. Y., Krier, J. D., Jordan, K. L., Textor, S. C., Grande, J. P., Lerman, A. and Lerman, L. O. (2010). Endothelial progenitor cells homing and renal repair in experimental renovascular disease. *Stem cells (Dayton, Ohio)* **28**, 1039.

Chade, A. R., Zhu, X., Lavi, R., Krier, J. D., Pislaru, S., Simari, R. D., Napoli, C., Lerman, A. and Lerman, L. O. (2009). Endothelial Progenitor Cells Restore Renal Function in Chronic Experimental Renovascular Disease. *Circulation* **119**, 547-557.

Challen, G. A., Bertoncello, I., Deane, J. A., Ricardo, S. D. and Little, M. H. (2006). Kidney Side Population Reveals Multilineage Potential and Renal Functional Capacity but also Cellular Heterogeneity. *Journal of the American Society of Nephrology* **17**, 1896-1912.

Chang, J.-W., Hung, S.-P., Wu, H.-H., Wu, W.-M., Yang, A.-H., Tsai, H.-L., Yang, L.-Y. and Lee, O. K. (2011). Therapeutic Effects of Umbilical Cord Blood-Derived Mesenchymal Stem Cell Transplantation in Experimental Lupus Nephritis. *Cell Transplantation* **20**, 245-257.

Chang, J. W., Tsai, H. L., Chen, C. W., Yang, H. W., Yang, A. H., Yang, L. Y., Wang, P. S., Ng, Y. Y., Lin, T. L. and Lee, O. K. (2012). Conditioned mesenchymal stem cells attenuate progression of chronic kidney disease through inhibition of epithelial-to-mesenchymal transition and immune modulation. *Journal of Cellular and Molecular Medicine* **16**, 2935.

Chen, J., Li, H., Addabbo, F., Zhang, F., Pelger, E., Patschan, D., Park, H.-C., Kuo, M.-C., Ni, J., Gobe, G. et al. (2009). Adoptive Transfer of Syngeneic Bone Marrow-Derived Cells in Mice with Obesity-Induced Diabetes: Selenoorganic Antioxidant Ebselen Restores Stem Cell Competence. *The American Journal of Pathology* **174**, 701.
Choi, S., Park, M., Kim, J., Hwang, S., Park, S. and Lee, Y. (2009). The role of mesenchymal stem cells in the functional improvement of chronic renal failure. *Stem Cells and Development* **18**, 521.

Chung, B. H., Lim, S. W., Doh, K. C., Piao, S. G., Heo, S. B. and Yang, C. W. (2013). Human Adipose Tissue Derived Mesenchymal Stem Cells Aggravate Chronic Cyclosporin Nephrotoxicity by the Induction of Oxidative Stress. *PLoS ONE* **8**, e59693.

De Chiara, L., Fagoonee, S., Ranghino, A., Bruno, S., Camussi, G., Tolosano, E., Silengo, L. and Altruda, F. (2014). Renal cells from spermatogonial germline stem cells protect against kidney injury. *Journal of the American Society of Nephrology* **25**, 316-328.

Donizetti-Oliveira, C., Semedo, P., Burgos-Silva, M., Cenedeze, M. A., Malheiros, D. M. A. C., Reis, M. A., Pacheco-Silva, A. and mara, N. O. S. (2012). Adipose Tissue-Derived Stem Cell Treatment Prevents Renal Disease Progression. *Cell Transplantation* **21**, 1727.

Du, T., Zou, X., Cheng, J., Wu, S., Zhong, L., Ju, G., Zhu, J., Liu, G., Zhu, Y. and Xia, S. (2013). Human Wharton's jelly-derived mesenchymal stromal cells reduce renal fibrosis through induction of native and foreign hepatocyte growth factor synthesis in injured tubular epithelial cells. *Stem Cell Research & Therapy* **4**, 59.

Ebrahimi, B., Li, Z., Eirin, A., Zhu, X.-Y., Textor, S. C. and Lerman, L. O. (2012). Addition of endothelial progenitor cells to renal revascularization restores medullary tubular oxygen consumption in swine renal artery stenosis. *American Journal of Physiology - Renal Physiology* **302**, F1478-F1485.

Eirin, A., Zhu, X. Y., Krier, J. D., Tang, H., Jordan, K. L., Grande, J. P., Lerman, A., Textor, S. C. and Lerman, L. O. (2012). Adipose tissue-derived mesenchymal stem cells improve revascularization outcomes to restore renal function in swine atherosclerotic renal artery stenosis. *Stem Cells* **30**, 1030.

Eirin, A., Zhang, X., Zhu, X. Y., Tang, H., Jordan, K. L., Grande, J. P., Dietz, A. B., Lerman, A., Textor, S. C. and Lerman, L. O. (2014). Renal vein cytokine release as an index of renal parenchymal inflammation in chronic experimental renal artery stenosis. *Nephrology Dialysis Transplantation* **29**, 274-282.

Eirin, A., Zhu, X.-Y., Li, Z., Ebrahimi, B., Zhang, X., Tang, H., Korsmo, M. J., Chade, A. R., Grande, J. P., Ward, C. J. et al. (2013). Endothelial Outgrowth Cells Shift Macrophage Phenotype and Improve Kidney Viability in Swine Renal Artery Stenosis. *Arteriosclerosis, Thrombosis, and Vascular Biology* **33**, 1006-1013.

Ezquer, F., Ezquer, M., Simon, V., Pardo, F., YaÃ±ez, A., Carpio, D. and Conget, P. (2009). Endovenous Administration of Bone Marrow-Derived Multipotent Mesenchymal

Stromal Cells Prevents Renal Failure in Diabetic Mice. *Biology of Blood and Marrow Transplantation* **15**, 1354.

Fang, Y., Tian, X., Bai, S., Fan, J., Hou, W., Tong, H. and Li, D. (2012). Autologous transplantation of adipose-derived mesenchymal stem cells ameliorates streptozotocin-induced diabetic nephropathy in rats by inhibiting oxidative stress, pro-inflammatory cytokines and the p38 MAPK signaling pathway. *Int J Mol Med* **30**, 85-92.

Franquesa, M., Herrero, E., Torras, J., Ripoll, E., Flaquer, M., Gomà, M., Lloberas, N., Anegon, I., Cruzado, J. M., Grinyó, J. M. et al. (2012). Mesenchymal stem cell therapy prevents interstitial fibrosis and tubular atrophy in a rat kidney allograft model. *Stem Cells and Development* **21**, 3125-3135.

Furuhashi, K., Tsuboi, N., Shimizu, A., Katsuno, T., Kim, H., Saka, Y., Ozaki, T., Sado, Y., Imai, E., Matsuo, S. et al. (2013). Serum-Starved Adipose-Derived Stromal Cells Ameliorate Crescentic GN by Promoting Immunoregulatory Macrophages. *Journal of the American Society of Nephrology* **24**, 587-603.

Gatti, S., Bruno, S., Deregibus, M. C., Sordi, A., Cantaluppi, V., Tetta, C. and Camussi, G. (2011). Microvesicles derived from human adult mesenchymal stem cells protect against ischaemia-reperfusion-induced acute and chronic kidney injury. *Nephrology Dialysis Transplantation*.

Gu, Z., Akiyama, K., Ma, X., Zhang, H., Feng, X., Yao, G., Hou, Y., Lu, L., Gilkeson, G., Silver, R. et al. (2010). Transplantation of umbilical cord mesenchymal stem cells alleviates lupus nephritis in MRL/lpr mice. *Lupus* **19**, 1502-1514.

Guo, J., Zou, Y., Wu, Z., Wu, W., Xu, Z., Hu, H., Huang, L., Dong, H., Chen, J., Lu, J. et al. (2014). Protective effects of mesenchymal stromal cells on adriamycin-induced minimal change nephrotic syndrome in rats and possible mechanisms. *Cytotherapy* **16**, 471-484.

Harrison, F., Yeagy, B. A., Rocca, C. J., Kohn, D. B., Salomon, D. R. and Cherqui, S. (2013). Hematopoietic stem cell gene therapy for the multisystemic lysosomal storage disorder cystinosis. *Molecular Therapy* **21**, 433-444.

He, J., Wang, Y., Sun, S., Yu, M., Wang, C., Pei, X., Zhu, B., Wu, J. and Zhao, W. (2012). Bone marrow stem cells-derived microvesicles protect against renal injury in the mouse remnant kidney model. *Nephrology* **17**, 493.

Huang, Z.-Y., Hong, L.-Q., Na, N., Luo, Y., Miao, B. and Chen, J. (2012). Infusion of mesenchymal stem cells overexpressing GDNF ameliorates renal function in nephrotoxic serum nephritis. *Cell Biochemistry and Function* **30**, 139.

Hyun, Y. Y., Kim, I. O., Kim, M. H., Nam, D. H., Lee, M. H., Kim, J. E., Song, H. K., Cha, J. J., Kang, Y. S., Lee, J. E. et al. (2012). Adipose-derived stem cells improve renal function in a mouse model of IgA nephropathy. *Cell Transplantation* **21**, 2425-2439.

Jiao, Y. Q., Yi, Z. W., He, X. J., Liu, X. H., He, Q. N. and Huang, D. L. (2011). Does injection of metanephric mesenchymal cells improve renal function in rats? *Saudi journal of kidney diseases and transplantation : an official publication of the Saudi Center for Organ Transplantation, Saudi Arabia* **22**, 501-510.

Kunter, U., Rong, S., Boor, P., Eitner, F., MÃ¼ller-Newen, G., Djuric, Z., Van Roeyen, C. R., Konieczny, A., Ostendorf, T., Villa, L. et al. (2007). Mesenchymal stem cells prevent

progressive experimental renal failure but maldifferentiate into glomerular adipocytes. *Journal of the American Society of Nephrology* **18**, 1754.

Lee, S. R., Lee, S. H., Moon, J. Y., Park, J. Y., Lee, D., Lim, S. J., Jeong, K. H., Park, J. K., Lee, T. W. and Ihm, C. G. (2010). Repeated administration of bone marrow-derived mesenchymal stem cells improved the protective effects on a remnant kidney model. *Renal Failure* **32**, 840.

Li, K., Han, Q., Yan, X., Liao, L. and Zhao, R. C. (2010). Not a process of simple vicariousness, the differentiation of human adipose-derived mesenchymal stem cells to renal tubular epithelial cells plays an important role in acute kidney injury repairing. *Stem Cells and Development* **19**, 1267-1275.

Li, W., Jiang, H. and Feng, J. M. (2012). Isogenic mesenchymal stem cells transplantation improves a rat model of chronic aristolochic acid nephropathy via upregulation of hepatic growth factor and downregulation of transforming growth factor Î²1. *Molecular and Cellular Biochemistry* **368**, 137.

Lv, S.-S., Liu, G., Wang, J.-P., Wang, W.-W., Cheng, J., Sun, A.-L., Liu, H.-Y., Nie, H.-B., Su, M.-R. and Guan, G.-J. (2013). Mesenchymal stem cells transplantation ameliorates glomerular injury in streptozotocin-induced diabetic nephropathy in rats via inhibiting macrophage infiltration. *International Immunopharmacology* **17**, 275-282.

Ma, H., Wu, Y., Xu, Y., Sun, L. and Zhang, X. (2013). Human umbilical mesenchymal stem cells attenuate the progression of focal segmental glomerulosclerosis. *American Journal of the Medical Sciences*.

Ma, H., Wu, Y., Zhang, W., Dai, Y., Li, F., Xu, Y., Wang, Y., Tu, H., Li, W. and Zhang, X. (2013). The effect of mesenchymal stromal cells on doxorubicin-induced nephropathy in rats. *Cytotherapy*.

Masoad, R. E., Ewais, M. M. S., Tawfik, M. K. and Abd El-All, H. S. (2012). Effect of mononuclear cells versus pioglitazone on streptozotocin-induced diabetic nephropathy in rats. *Pharmacological Reports* **64**, 1223-1233.

Ninichuk, V., Gross, O., Segerer, S., Hoffmann, R., Radomska, E., Buchstaller, A., Huss, R., Akis, N., SchlÃ¶ndorff, D. and Anders, H. J. (2006). Multipotent mesenchymal stem cells reduce interstitial fibrosis but do not delay progression of chronic kidney disease in collagen4A3-deficient mice. *Kidney International* **70**, 121.

Nur, R., Fukuda, N., Matsumoto, T., Medet, J., Kano, K., Yamamoto, C., Maruyama, T., Endo, M. and Matsumoto, K. (2008). Implantation of dedifferentiated fat cells ameliorates habu snake venom-induced chronic renal dysfunction in tenascin-C-deficient mice. *Nephron - Experimental Nephrology* **110**, e91-e98.

Oliveira-Sales, E. B., Maquigussa, E., Semedo, P., Pereira, L. G., Ferreira, V. M., Câmara, N. O., Bergamaschi, C. T., Campos, R. R. and Boim, M. A. (2013). Mesenchymal Stem Cells (MSC) Prevented the Progression of Renovascular Hypertension, Improved Renal Function and Architecture. *PLoS ONE* **8**, e78464.

Park, J. H., Park, J., Hwang, S. H., Han, H. and Ha, H. (2012). Delayed Treatment With Human Umbilical Cord Blood-Derived Stem Cells Attenuates Diabetic Renal Injury. *Transplantation Proceedings* **44**, 1123.

Park, J. H., Hwang, I., Hwang, S. H., Han, H. and Ha, H. (2012). Human umbilical cord blood-derived mesenchymal stem cells prevent diabetic renal injury through paracrine action. *Diabetes Research and Clinical Practice* **98**, 465.

Ratliff, B. B., Ghaly, T., Brudnicki, P., Yasuda, K., Rajdev, M., Bank, M., Mares, J., Hatzopoulos, A. K. and Goligorsky, M. S. (2010). Endothelial progenitors encapsulated in bioartificial niches are insulated from systemic cytotoxicity and are angiogenesis competent. *American Journal of Physiology - Renal Physiology* **299**, F178.

Sangidorj, O., Yang, S. H., Jang, H. R., Lee, J. P., Cha, R. H., Kim, S. M., Lim, C. S. and Kim, Y. S. (2010). Bone marrow-derived endothelial progenitor cells confer renal protection in a murine chronic renal failure model. *American Journal of Physiology - Renal Physiology* **299**.

Semedo, P., Donizetti-Oliveira, C., Burgos-Silva, M., Cenedeze, M. A., Avancini Costa Malheiros, D. M., Pacheco-Silva, A. and Camara, N. O. S. (2010). Bone marrow mononuclear cells attenuate fibrosis development after severe acute kidney injury. *Laboratory Investigation* **90**, 685.

Semedo, P., Correa-Costa, M., Cenedeze, M. A., Malheiros, D. M. A. C., Dos Reis, M. A., Shimizu, M. H., Seguro, A. C., Pacheco-Silva, A. and Äˆamara, N. O. S. (2009). Mesenchymal stem cells attenuate renal fibrosis through immune modulation and remodeling properties in a rat remnant kidney model. *Stem Cells* **27**, 3063.

Shuai, L., Li, X., He, Q., Dang, X., Chen, H., Zhou, P., Yi, Z. and He, X. (2012). Angiogenic Effect of Endothelial Progenitor Cells Transfected with Telomerase Reverse Transcriptase on Peritubular Microvessel in Five Out of Six Subtotal Nephrectomy Rats. *Renal Failure* **34**, 1270-1280.

Togel, F., Cohen, A., Zhang, P., Yang, Y., Hu, Z. and Westenfelder, C. (2009). Autologous and allogeneic marrow stromal cells are safe and effective for the treatment of acute kidney injury. *Stem Cells and Development* **18**, 475.

Urbieta-Caceres, V. H., Zhu, X.-Y., Jordan, K. L., Tang, H., Textor, K., Lerman, A. and Lerman, L. O. (2012). Selective improvement in renal function preserved remote myocardial microvascular integrity and architecture in experimental renovascular disease. *Atherosclerosis* **221**, 350.

van Koppen, A., Joles, J. A., van Balkom, B. W. M., Lim, S. K., de Kleijn, D., Giles, R. H. and Verhaar, M. C. (2012a). Human embryonic mesenchymal stem cell-derived conditioned medium rescues kidney function in rats with established chronic kidney disease. *PLoS ONE* 7(6), e38746. doi:10.1371/journal.pone.0038746

van Koppen, A., Joles, J. A., Bongartz, L. G., van den Brandt, J., Reichardt, H. M., Goldschmeding, R., Nguyen, T. Q. and Verhaar, M. C. (2012b). Healthy Bone Marrow Cells Reduce Progression Of Kidney Failure Better Than CKD Bone Marrow Cells In Rats With Established Chronic Kidney Disease. *Cell Transplant*. **21**, 2299–2312.

Villanueva, S., Ewertz, E., Carrión, F., Tapia, A., Vergara, C., Céspedes, C., Sáez, P. J., Luz, P., Irarrázabal, C., Carreño, J. E. et al. (2011). Mesenchymal stem cell injection ameliorates chronic renal failure in a rat model. *Clinical Science* **121**, 489-499.

Villanueva, S., Carreño, J. E., Salazar, L., Vergara, C., Strodthoff, R., Fajre, F., Céspedes, C., Sáez, P. J., Irarrázabal, C., Bartolucci, J. et al. (2013). Human

mesenchymal stem cells derived from adipose tissue reduce functional and tissue damage in a rat model of chronic renal failure. *Clinical Science* **125**, 199-210.

Wang, S., Li, Y., Zhao, J., Zhang, J. and Huang, Y. (2013). Mesenchymal Stem Cells Ameliorate Podocyte Injury and Proteinuria in a Type 1 Diabetic Nephropathy Rat Model. *Biology of Blood and Marrow Transplantation* **19**, 538.

Yamaleyeva, L. M., Guimaraes-Souza, N. K., Krane, L. S., Agcaoili, S., Gyabaah, K., Atala, A., Aboushwareb, T. and Yoo, J. J. (2012). Cell Therapy with Human Renal Cell Cultures Containing Erythropoietin-Positive Cells Improves Chronic Kidney Injury. *Stem Cells Translational Medicine* **1**, 373-383.

Yuen, D. A., Connelly, K. A., Advani, A., Liao, C., Kuliszewski, M. A., Trogadis, J., Thai, K., Advani, S. L., Zhang, Y., Kelly, D. J. et al. (2010). Culture-modified bone marrow cells attenuate cardiac and renal injury in a chronic kidney disease rat model via a novel antifibrotic mechanism. *PLoS ONE* **5**.

Yuen, D. A., Connelly, K. A., Zhang, Y., Advani, S. L., Thai, K., Kabir, G., Kepecs, D., Spring, C., Smith, C., Batruch, I. et al. (2013). Early outgrowth cells release soluble endocrine antifibrotic factors that reduce progressive organ fibrosis. *Stem Cells* **31**, 2408-2419.

Zhang, L., Li, K., Liu, X., Li, D., Luo, C., Fu, B., Cui, S., Zhu, F., Zhao, R. C. and Chen, X. (2013). Repeated systemic administration of human adipose-derived stem cells attenuates overt diabetic nephropathy in rats. *Stem Cells Dev* **22**, 3074-3086.

Zhang, Y., Yuen, D. A., Advani, A., Thai, K., Advani, S. L., Kepecs, D., Kabir, M. G., Connelly, K. A. and Gilbert, R. E. (2012). Early-Outgrowth Bone Marrow Cells Attenuate Renal Injury and Dysfunction via an Antioxidant Effect in a Mouse Model of Type 2 Diabetes. *Diabetes* **61**, 2114-2125.

Zhang, Y., Ye, C., Wang, G., Gao, Y., Tan, K., Zhuo, Z., Liu, Z., Xia, H., Yang, D. and Li, P. (2013). Kidney-Targeted Transplantation of Mesenchymal Stem Cells by Ultrasound-Targeted Microbubble Destruction Promotes Kidney Repair in Diabetic Nephropathy Rats. *BioMed Research International* **2013**, 13.

Zhou, H., Tian, H. M., Long, Y., Zhang, X. X., Zhong, L., Deng, L., Chen, X. H. and Li, X. Q. (2009). Mesenchymal stem cells transplantation mildly ameliorates experimental diabetic nephropathy in rats. *Chinese Medical Journal* **122**, 2573.

Zhu, X.-Y., Urbieta-Caceres, V., Krier, J. D., Textor, S. C., Lerman, A. and Lerman, L. O. (2013). Mesenchymal Stem Cells and Endothelial Progenitor Cells Decrease Renal Injury in Experimental Swine Renal Artery Stenosis Through Different Mechanisms. *STEM CELLS* **31**, 117.

Zoja, C., Garcia, P. B., Rota, C., Conti, S., Gagliardini, E., Corna, D., Zanchi, C., Bigini, P., Benigni, A., Remuzzi, G. et al. (2012). Mesenchymal stem cell therapy promotes renal repair by limiting glomerular podocyte and progenitor cell dysfunction in adriamycin-induced nephropathy. *American Journal of Physiology - Renal Physiology* **303**, F1370-F1381.

Table S2. Pooled cell types.

BM	*EPC*	*MSC*	*HSC*	*Embryonic*	*Organ specific*	*Cell product*	*Other*
BM	BM CMC	ADSC	HSPC	DFAT	GTC	EOC CM	BM-M0
BMMNC	EOC	chMSC	Lin^{-} BM		hPKC	EPC MV	BM-M2
hUB-MNC	EPC	hADMSC	Lin^{-}CD90^{+}HSC		MMC	hMSC CM	SP-M0
		hADSC			MP	hMSC Exo	SP-M2
		hHASC			SP	hMSC MV	
		hLASC				hU-MSC supernatant	
		hUB-MSC				MSC MV	
		hUB-SC					
		hU-MSC					
		hWJ-MSC					
		MSC					
		SC					

Abbreviations: ADSC, adipose-tissue derived stem cell; BM, bone marrow cells; BMMNC, bone marrow mononuclear cells; BM-CMC, bone marrow-derived culture modified cells; BM-M0, bone marrow derived macrophage; BM-M2, bone marrow derived activated macrophage; chMSC, conditioned human mesenchymal stem cell; DFAT, dedifferentiated fat cells; EOC, endothelial outgrowth cell; EOC CM; endothelial outgrowth cell derived conditioned medium; EPC, endothelial progenitor cell; EPC MV, endothelial progenitor cell derived micro vesicle; GTC , germline cell derived tubular-like cells; hPKC, human primary kidney cells; hUB-MNC, human umbilical cord blood mononuclear cell; hU-MSC, human umbilical cord MSC; hADMSC, human adipose tissue derived mesenchymal stem cell; hADSC, human adipose tissue derived stem cell; hHASC, human high serum level cultured adipose-tissue derived stromal cells; hLASC, human low serum level cultured adipose-tissue derived stromal cells; hMSC CM, human mesenchymal stem cell derived conditioned medium; hMSC Exo, human MSC derived exosomes; hMSC MV, human mesenchymal stem cell derived microvesicle; HSPC, hematopoietic stem and progenitor cell; hUB-MSC, human umbilical cord blood derived mesenchymal stem cell; hUB-SC, human umbilical cord blood derived stem cell; hWJ-MSC, human wharton’s jelly derived mesenchymal stem cell; Lin^{-} BM, lineage negative bone marrow cells; Lin^{-}CD90^{+}HSC, lineage negative CD90 positive hematopoietic stem cell, MMC, metanephric mesenchymal cell; MP, main population kidney cells; MSC, mesenchymal stem cell; MSC MV, MSC derived micro vesicles; SC, stromal cell; SP, side population kidney cells; SP-M0, spleen derived macrophage; SP-M2, spleen derived activated macrophage.

Table S3. Pooled models.

Subtotal nephrectomy	***Ischemia reperfusion***	***Diabetes***	***Toxic***	***Genetic non-diabetes***	***Hypertension***
2/3 Nx	IRI	DN (Db/Db)	Adriamycin	Alport syndrome	2K-1C
5/6 Nx	IRI + gentamicin	DN	Anti-GBM GN	Cystinosis	RAS
	Unx + IRI		CAAN	IgAN	RAS + PTRA
	Unx + IRI + CsA		CAN	Lupus nephritis	
			CsA		
			HSV		
			NSN		
			Unx + aThy		

Abbreviations: 2K-1C, two kidney-one clip; Anti-GBM GN, anti glomerular basement membrane glomerulonephritis; aThy; anti-Thy-1; CAN, chronic allograft nephropathy; CAAN, chronic aristolochic acid nephropathy; CsA, cyclosporine; DN, diabetic nephropathy; HSV, habu snake venom; IgAN, IgA nephropathy; IRI, ischemia reperfusion injury; NSN, nephrotoxic serum nephritis; Nx, nephrectomy; Unx, uninephrectomy; RAS, renal artery stenosis; PTRA, percutaneous transluminal renal angioplasty.

Fig. S1. Effect of cell-based treatment in CKD on plasma creatinine.

Forest plot, right side shows improvement by cell-based therapy. Data are presented as SMDs and 95% CI

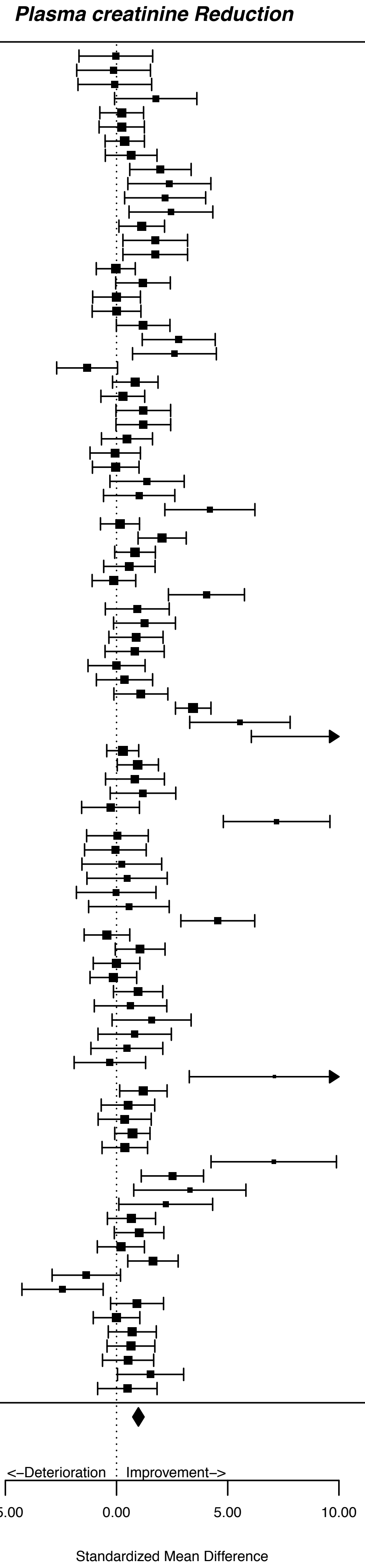

Author and Year	*Animal*	*Cell Type*	*SMD & 95% CI*
Cao Q (2013)	mouse	Other	–0.03 [–1.69 , 1.62]
Cao Q (2013)	mouse	Other	–0.13 [–1.79 , 1.53]
Cao Q (2013)	mouse	Other	–0.08 [–1.73 , 1.58]
Cao Q (2013)	mouse	Other	1.76 [–0.09 , 3.61]
Chang JW (2011)	mouse	MSC	0.23 [–0.75 , 1.22]
Chang JW (2011)	mouse	MSC	0.23 [–0.78 , 1.25]
De Chiara L (2013)	mouse	Organ specific	0.37 [–0.51 , 1.25]
Donizettie–Oliveira C (2012)	mouse	MSC	0.66 [–0.51 , 1.82]
Donizettie–Oliveira C (2012)	mouse	MSC	1.97 [0.59 , 3.35]
Gu Z (2010)	mouse	MSC	2.38 [0.51 , 4.24]
Gu Z (2010)	mouse	MSC	2.19 [0.36 , 4.01]
Gu Z (2010)	mouse	MSC	2.45 [0.56 , 4.33]
Harrison F (2013)	mouse	HSC	1.13 [0.11 , 2.16]
He J (2012)	mouse	MSC	1.74 [0.29 , 3.19]
He J (2012)	mouse	Cell product	1.74 [0.29 , 3.19]
Ninichuk V (2006)	mouse	MSC	–0.03 [–0.91 , 0.84]
Ratliff BB (2010)	mouse	EPC	1.19 [–0.04 , 2.42]
Sangidorj O (2010)	mouse	EPC	0.00 [–1.07 , 1.07]
Sangidorj O (2010)	mouse	EPC	0.00 [–1.10 , 1.10]
Sangidorj O (2010)	mouse	EPC	1.20 [–0.01 , 2.40]
Sangidorj O (2010)	mouse	EPC	2.80 [1.16 , 4.44]
Sangidorj O (2010)	mouse	EPC	2.60 [0.72 , 4.49]
Semedo P (2010)	mouse	BM	–1.32 [–2.69 , 0.05]
Alfarano C (2012)	rat	MSC	0.84 [–0.18 , 1.86]
Alfarano C (2012)	rat	MSC	0.28 [–0.70 , 1.27]
Bian X (2014)	rat	MSC	1.20 [–0.03 , 2.43]
Bian X (2014)	rat	MSC	1.20 [–0.03 , 2.43]
Bian X (2014)	rat	MSC	0.47 [–0.68 , 1.62]
Bian X (2014)	rat	MSC	–0.06 [–1.19 , 1.07]
Burst V (2013)	rat	HSC	–0.04 [–1.08 , 1.01]
Caldas HC (2008)	rat	BM	1.37 [–0.30 , 3.04]
Caldas HC (2008)	rat	MSC	1.02 [–0.59 , 2.62]
Cantaluppi V (2012)	rat	Cell product	4.20 [2.17 , 6.22]
Castiglione RC (2013)	rat	BM	0.16 [–0.72 , 1.03]
Cavaglieri RC (2009)	rat	MSC	2.05 [0.97 , 3.13]
Cavaglieri RC (2009)	rat	MSC	0.83 [–0.08 , 1.75]
Chang JW (2012)	rat	MSC	0.58 [–0.58 , 1.73]
Chung BH (2013)	rat	MSC	–0.12 [–1.10 , 0.86]
Fang Y (2012)	rat	MSC	4.04 [2.33 , 5.75]
Franquesa M (2012)	rat	BM	0.93 [–0.50 , 2.37]
Franquesa M (2012)	rat	MSC	1.26 [–0.13 , 2.65]
Furuhashi K (2013)	rat	MSC	0.87 [–0.34 , 2.09]
Furuhashi K (2013)	rat	MSC	0.81 [–0.52 , 2.14]
Furuhashi K (2013)	rat	MSC	0.00 [–1.28 , 1.28]
Furuhashi K (2013)	rat	MSC	0.36 [–0.91 , 1.62]
Gatti S (2011)	rat	Cell product	1.09 [–0.12 , 2.31]
Guo J (2013)	rat	MSC	3.45 [2.65 , 4.25]
Huang ZY (2012)	rat	MSC	5.55 [3.29 , 7.81]
Huang ZY (2012)	rat	MSC	9.69 [6.06 , 13.32]
Jiao YQ (2011)	rat	Organ specific	0.28 [–0.44 , 1.00]
Kunter U (2007)	rat	MSC	0.96 [0.03 , 1.88]
Lee SR (2010)	rat	MSC	0.83 [–0.49 , 2.15]
Lee SR (2010)	rat	MSC	1.19 [–0.29 , 2.66]
Lee SR (2010)	rat	Organ specific	–0.27 [–1.57 , 1.03]
Li W (2012)	rat	MSC	7.20 [4.80 , 9.60]
Ma H (2013)	rat	MSC	0.04 [–1.35 , 1.43]
Ma H (2013)	rat	MSC	–0.05 [–1.44 , 1.33]
Ma H (2013)	rat	MSC	0.24 [–1.56 , 2.03]
Ma H (2013)	rat	MSC	0.47 [–1.33 , 2.28]
Ma H (2013)	rat	MSC	–0.02 [–1.81 , 1.77]
Ma H (2013)	rat	Cell product	0.56 [–1.25 , 2.37]
Masoad RE (2012)	rat	BM	4.55 [2.89 , 6.22]
Oliveira–Sales EB (2013)	rat	MSC	–0.43 [–1.46 , 0.60]
Park JH (2012)	rat	MSC	1.06 [–0.06 , 2.18]
Park JH (2012)	rat	MSC	0.00 [–1.05 , 1.05]
Semedo P (2009)	rat	MSC	–0.14 [–1.19 , 0.91]
Semedo P (2009)	rat	MSC	0.97 [–0.14 , 2.08]
Toegel F (2009)	rat	MSC	0.63 [–1.00 , 2.26]
Toegel F (2009)	rat	MSC	1.58 [–0.20 , 3.35]
Toegel F (2009)	rat	MSC	0.82 [–0.83 , 2.46]
Toegel F (2009)	rat	MSC	0.46 [–1.15 , 2.08]
Toegel F (2009)	rat	MSC	–0.30 [–1.91 , 1.31]
Toegel F (2009)	rat	MSC	7.10 [3.27 , 10.93]
van Koppen A (2012)	rat	BM	1.21 [0.14 , 2.27]
van Koppen A (2012)	rat	BM	0.51 [–0.69 , 1.72]
van Koppen A (2012)	rat	BM	0.37 [–0.83 , 1.56]
van Koppen A (2012)	rat	Cell product	0.71 [–0.08 , 1.50]
van Koppen A (2012)	rat	Cell product	0.37 [–0.65 , 1.40]
Villanueva S (2013)	rat	MSC	7.07 [4.25 , 9.89]
Villanueva S (2011)	rat	MSC	2.51 [1.11 , 3.91]
Yamaleyeva LM (2012)	rat	Organ specific	3.30 [0.77 , 5.82]
Yamaleyeva LM (2012)	rat	Organ specific	2.21 [0.10 , 4.32]
Yuen DA (2010)	rat	EPC	0.67 [–0.41 , 1.75]
Yuen DA (2010)	rat	EPC	1.01 [–0.10 , 2.13]
Yuen DA (2010)	rat	MSC	0.19 [–0.86 , 1.25]
Zhang L (2012)	rat	MSC	1.64 [0.51 , 2.77]
Zoja C (2012)	rat	MSC	–1.36 [–2.90 , 0.18]
Zoja C (2012)	rat	MSC	–2.43 [–4.25 , –0.60]
Eirin A (2014)	pig	MSC	0.92 [–0.27 , 2.11]
Eirin A (2013)	pig	EPC	0.00 [–1.05 , 1.05]
Eirin A (2013)	pig	EPC	0.71 [–0.37 , 1.79]
Eirin A (2012)	pig	MSC	0.65 [–0.43 , 1.72]
Urbieta–Caceres VH (2012)	pig	EPC	0.52 [–0.63 , 1.67]
Zhu XY (2013)	pig	MSC	1.53 [0.04 , 3.02]
Zhu XY (2013)	pig	EPC	0.49 [–0.85 , 1.82]
RE Model			0.98 [0.73 , 1.24]

p–value = 3.23e–14

τ = 1.04

I^2 = 72.96%

Fig. S2. Effect of cell-based treatment in CKD on glomerular filtration rate (GFR).
Forest plot, right side shows improvement by cell-based thereapy. Data are presented as SMDs and 95% CI.

Author and Year	*Animal*	*Cell Type*	*Method*	*SMD & 95% CI*
Cao Q (2013)	mouse	Other	Creatinine	0.20 [−1.46 , 1.86]
Cao Q (2013)	mouse	Other	Creatinine	0.00 [−1.66 , 1.66]
Cao Q (2013)	mouse	Other	Creatinine	0.07 [−1.59 , 1.72]
Cao Q (2013)	mouse	Other	Creatinine	1.40 [−0.38 , 3.18]
Harrison F (2013)	mouse	HSC	Creatinine	0.23 [−0.72 , 1.19]
Alexandre CS (2009)	rat	HSC	Inulin	0.20 [−1.00 , 1.41]
Alexandre CS (2009)	rat	HSC	Inulin	0.00 [−1.20 , 1.20]
Alexandre CS (2009)	rat	HSC	Inulin	1.80 [0.71 , 2.89]
Caldas HC (2011)	rat	MSC	Creatinine	1.42 [−0.26 , 3.10]
Caldas HC (2011)	rat	BM	Creatinine	0.79 [−0.78 , 2.36]
Caldas HC (2011)	rat	MSC	Creatinine	1.16 [−0.47 , 2.79]
Caldas HC (2011)	rat	BM	Creatinine	1.51 [−0.19 , 3.21]
Caldas HC (2008)	rat	BM	Creatinine	1.07 [−0.54 , 2.68]
Caldas HC (2008)	rat	MSC	Creatinine	0.11 [−1.41 , 1.62]
Castiglione RC (2013)	rat	BM	Creatinine	−0.59 [−1.49 , 0.31]
Chang JW (2012)	rat	MSC	Creatinine	2.08 [0.67 , 3.48]
Chung BH (2013)	rat	MSC	Creatinine	−0.08 [−1.06 , 0.90]
Huang ZY (2012)	rat	MSC	Creatinine	2.23 [0.89 , 3.56]
Huang ZY (2012)	rat	MSC	Creatinine	4.38 [2.48 , 6.28]
Jiao YQ (2011)	rat	Organ specific	Creatinine	1.17 [0.39 , 1.94]
Kunter U (2007)	rat	MSC	Creatinine	−0.36 [−1.25 , 0.52]
Lv SS (2013)	rat	MSC	Creatinine	−1.82 [−2.64 , −1.00]
Semedo P (2009)	rat	MSC	Inulin	1.10 [−0.03 , 2.22]
Shuai L (2012)	rat	EPC	Creatinine	2.08 [0.44 , 3.73]
Shuai L (2012)	rat	EPC	Creatinine	3.99 [1.80 , 6.18]
Shuai L (2012)	rat	EPC	Creatinine	4.74 [2.30 , 7.18]
Shuai L (2012)	rat	EPC	Creatinine	3.23 [1.28 , 5.18]
Shuai L (2012)	rat	EPC	Creatinine	2.88 [1.03 , 4.72]
Shuai L (2012)	rat	EPC	Creatinine	6.00 [3.10 , 8.90]
Shuai L (2012)	rat	EPC	Creatinine	7.43 [3.98 , 10.87]
Shuai L (2012)	rat	EPC	Creatinine	4.75 [2.30 , 7.20]
Shuai L (2012)	rat	EPC	Creatinine	10.76 [5.99 , 15.53]
Toegel F (2009)	rat	MSC	Creatinine	0.81 [−0.37 , 1.99]
Toegel F (2009)	rat	MSC	Creatinine	0.43 [−1.19 , 2.04]
Toegel F (2009)	rat	MSC	Creatinine	0.27 [−1.34 , 1.87]
Toegel F (2009)	rat	MSC	Creatinine	0.11 [−1.49 , 1.71]
Toegel F (2009)	rat	MSC	Creatinine	0.41 [−1.20 , 2.03]
Toegel F (2009)	rat	MSC	Creatinine	−0.48 [−2.09 , 1.14]
Toegel F (2009)	rat	MSC	Creatinine	2.89 [0.76 , 5.03]
van Koppen A (2012)	rat	BM	Inulin	1.38 [0.29 , 2.47]
van Koppen A (2012)	rat	BM	Inulin	1.10 [−0.16 , 2.37]
van Koppen A (2012)	rat	BM	Inulin	0.13 [−1.05 , 1.32]
van Koppen A (2012)	rat	Cell product	Inulin	1.08 [0.25 , 1.90]
van Koppen A (2012)	rat	Cell product	Inulin	−0.09 [−1.11 , 0.92]
Wang S (2013)	rat	MSC	Creatinine	−4.48 [−6.26 , −2.70]
Yuen DA (2013)	rat	EPC	Inulin	1.08 [−0.15 , 2.31]
Yuen DA (2013)	rat	Cell product	Inulin	1.15 [−0.06 , 2.36]
Zhang L (2012)	rat	MSC	Creatinine	1.42 [0.32 , 2.51]
Zhou H (2009)	rat	MSC	Creatinine	−0.43 [−2.14 , 1.29]
Zhou H (2009)	rat	MSC	Creatinine	−0.46 [−2.18 , 1.26]
Chade AR (2010)	pig	EPC	MDCT	1.25 [0.10 , 2.39]
Chade AR (2009)	pig	EPC	MDCT	0.73 [−0.36 , 1.81]
Ebrahimi B (2012)	pig	EPC	MDCT	0.33 [−0.72 , 1.39]
Eirin A (2014)	pig	MSC	MDCT	1.89 [0.53 , 3.25]
Eirin A (2013)	pig	EPC	MDCT	0.93 [−0.17 , 2.03]
Eirin A (2013)	pig	EPC	MDCT	0.51 [−0.56 , 1.57]
Eirin A (2012)	pig	MSC	MDCT	0.53 [−0.53 , 1.60]
Urbieta−Caceres VH (2012)	pig	EPC	MDCT	0.91 [−0.28 , 2.09]
Zhu XY (2013)	pig	MSC	MDCT	2.18 [0.54 , 3.82]
Zhu XY (2013)	pig	EPC	MDCT	0.89 [−0.49 , 2.27]
RE Model				1.05 [0.67 , 1.43]

p−value = 7.27 e−08
τ =1.29
I^2 = 79.1%

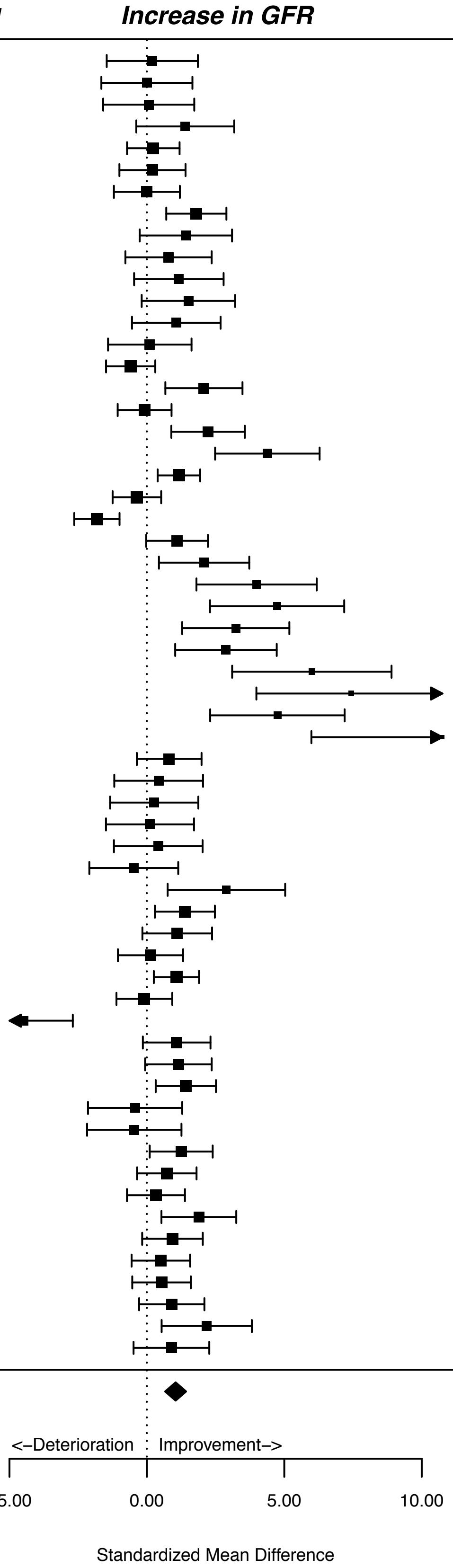

Fig. S3. Correlations between renal functional parameters and tissue injury parameters.
Decrease in urinary protein vs. decrease in glomerulosclerosis, GS (A);
decrease in urinary protein vs. decrease in intersitial fibrosis, IF (B);
decrease in plasma creatinine vs. decrease in GS (C);
decrease in plasma creatinine vs. decrease in IF (D);
increase in glomerular filtration rate, GFR vs. decrease in GS (E); increase in GFR vs. decrease in IF (F).

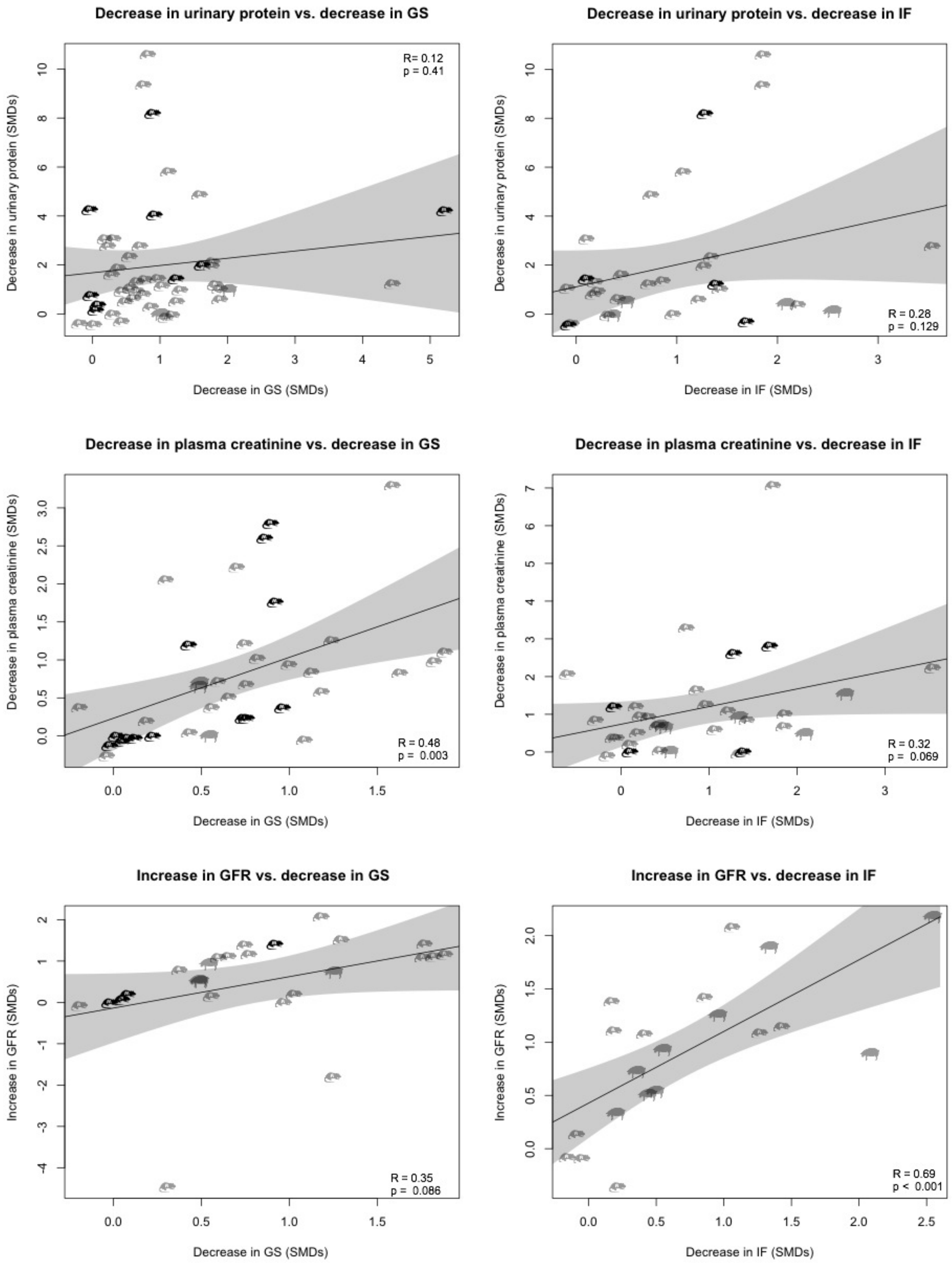

Fig. S4. Subgroup analysis of cell-based treatment related factors. Plasma creatinine (A), plasma urea (B), glomerular filtration rate, GFR (C), blood pressure, BP (D), urinary protein (E), glomerulosclerosis, GS (F) and interstitial fibrosis, IF (G). Right side shows improvement by cell-based therapy. Data are presented as SMDs and 95% Cl.

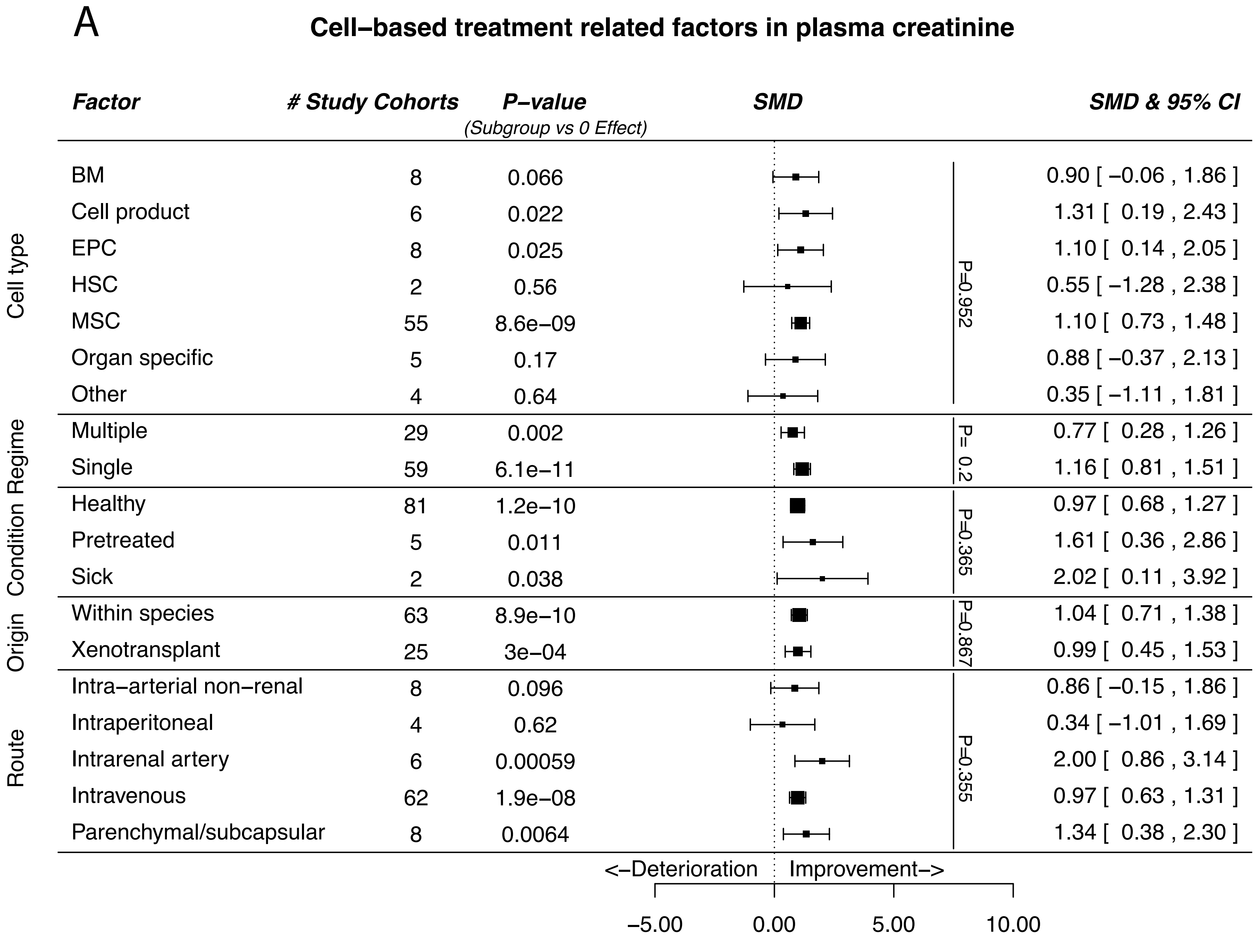

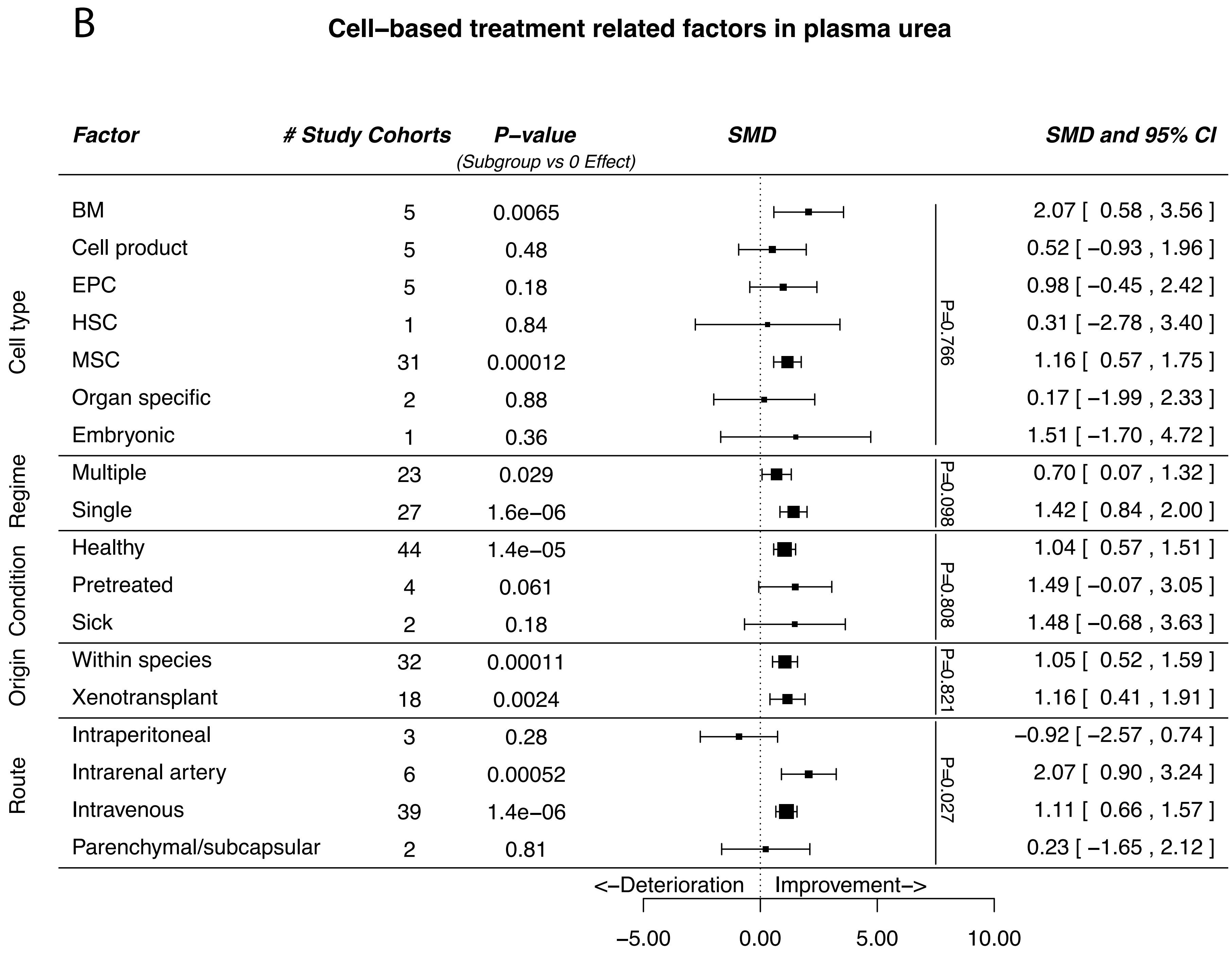
B
Cell–based treatment related factors in plasma urea
Factor
Study Cohorts
P–value
(Subgroup vs 0 Effect)
SMD
SMD and 95% CI
Cell type
BM 5 0.0065 2.07 [0.58 , 3.56]
Cell product 5 0.48 0.52 [−0.93 , 1.96]
EPC 5 0.18 0.98 [−0.45 , 2.42]
HSC 1 0.84 0.31 [−2.78 , 3.40]
MSC 31 0.00012 1.16 [0.57 , 1.75]
Organ specific 2 0.88 0.17 [−1.99 , 2.33]
Embryonic 1 0.36 1.51 [−1.70 , 4.72]
P=0.766
Regime
Multiple 23 0.029 0.70 [0.07 , 1.32]
Single 27 1.6e−06 1.42 [0.84 , 2.00]
P=0.098
Condition
Healthy 44 1.4e−05 1.04 [0.57 , 1.51]
Pretreated 4 0.061 1.49 [−0.07 , 3.05]
Sick 2 0.18 1.48 [−0.68 , 3.63]
P=0.808
Origin
Within species 32 0.00011 1.05 [0.52 , 1.59]
Xenotransplant 18 0.0024 1.16 [0.41 , 1.91]
P=0.821
Route
Intraperitoneal 3 0.28 −0.92 [−2.57 , 0.74]
Intrarenal artery 6 0.00052 2.07 [0.90 , 3.24]
Intravenous 39 1.4e−06 1.11 [0.66 , 1.57]
Parenchymal/subcapsular 2 0.81 0.23 [−1.65 , 2.12]
P=0.027
<−Deterioration Improvement−>
−5.00 0.00 5.00 10.00

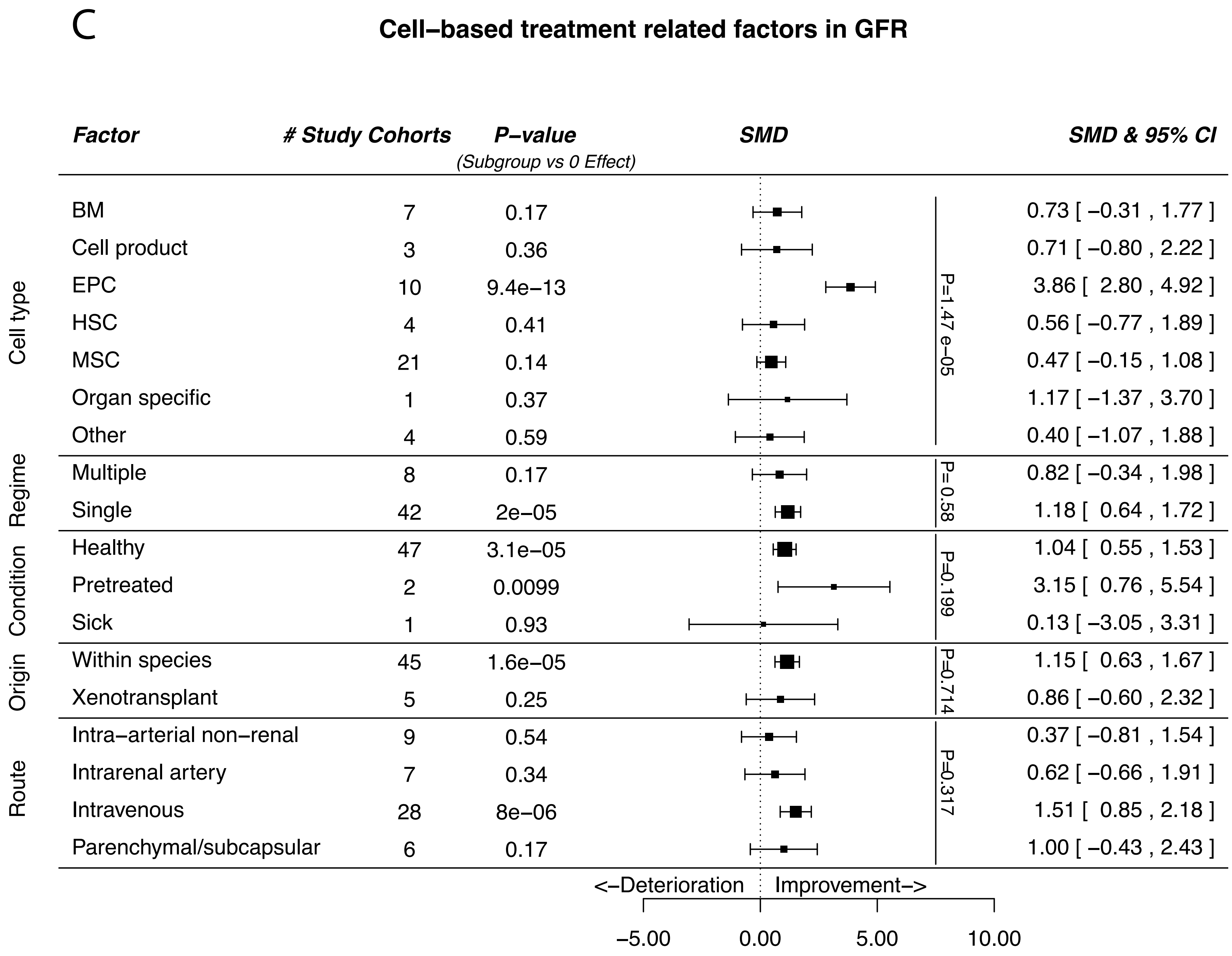
C
Cell–based treatment related factors in GFR
Factor
Study Cohorts
P–value
(Subgroup vs 0 Effect)
SMD
SMD & 95% CI
Cell type
BM 7 0.17 0.73 [–0.31 , 1.77]
Cell product 3 0.36 0.71 [–0.80 , 2.22]
EPC 10 9.4e–13 3.86 [2.80 , 4.92]
HSC 4 0.41 0.56 [–0.77 , 1.89]
MSC 21 0.14 0.47 [–0.15 , 1.08]
Organ specific 1 0.37 1.17 [–1.37 , 3.70]
Other 4 0.59 0.40 [–1.07 , 1.88]
P=1.47 e–05
Regime
Multiple 8 0.17 0.82 [–0.34 , 1.98]
Single 42 2e–05 1.18 [0.64 , 1.72]
P= 0.58
Condition
Healthy 47 3.1e–05 1.04 [0.55 , 1.53]
Pretreated 2 0.0099 3.15 [0.76 , 5.54]
Sick 1 0.93 0.13 [–3.05 , 3.31]
P=0.199
Origin
Within species 45 1.6e–05 1.15 [0.63 , 1.67]
Xenotransplant 5 0.25 0.86 [–0.60 , 2.32]
P=0.714
Route
Intra–arterial non–renal 9 0.54 0.37 [–0.81 , 1.54]
Intrarenal artery 7 0.34 0.62 [–0.66 , 1.91]
Intravenous 28 8e–06 1.51 [0.85 , 2.18]
Parenchymal/subcapsular 6 0.17 1.00 [–0.43 , 2.43]
P=0.317
<–Deterioration
Improvement–>
–5.00
0.00
5.00
10.00

D

Cell–based treatment related factors in BP

	Factor	***# Study Cohorts***	***P–value*** *(Subgroup vs 0 Effect)*	***SMD & 95% CI***	
Cell type	BM	4	0.00032	1.55 [0.70 , 2.39]	P=0.564
	Cell product	3	0.09	0.76 [−0.12 , 1.65]	
	EPC	3	0.19	0.62 [−0.31 , 1.56]	
	MSC	8	0.001	0.97 [0.39 , 1.54]	
	Organ specific	1	0.67	0.39 [−1.36 , 2.13]	
Regime	Multiple	5	0.007	0.99 [0.27 , 1.71]	P=0.937
	Single	14	1.7e−05	0.95 [0.52 , 1.39]	
Condition	Healthy	18	4.2e−07	0.98 [0.60 , 1.36]	P=0.701
	Sick	1	0.45	0.64 [−1.02 , 2.31]	
Origin	Within species	16	1.9e−05	0.88 [0.48 , 1.28]	P=0.328
	Xenotransplant	3	0.0023	1.36 [0.48 , 2.23]	
Route	Intra–arterial non–renal	2	0.55	0.31 [−0.71 , 1.34]	P=0.143
	Intrarenal artery	4	0.052	0.72 [0.00 , 1.44]	
	Intravenous	11	2.2e−05	0.97 [0.52 , 1.42]	
	Parenchymal/subcapsular	2	0.00019	1.92 [0.91 , 2.93]	

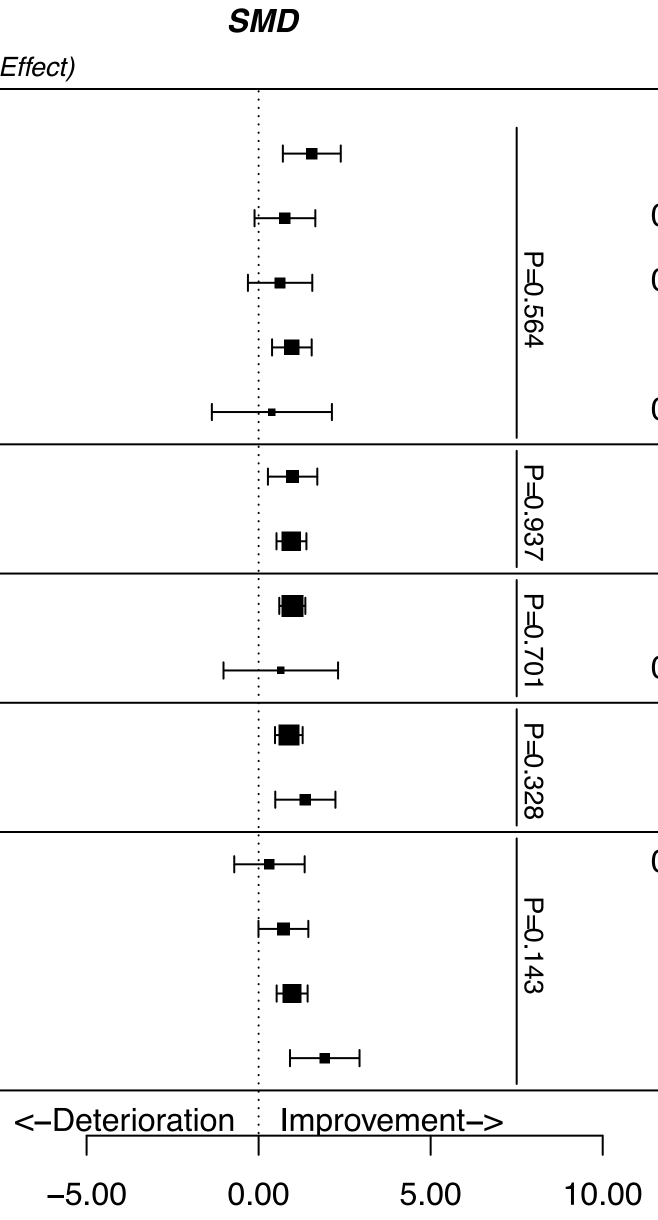

E

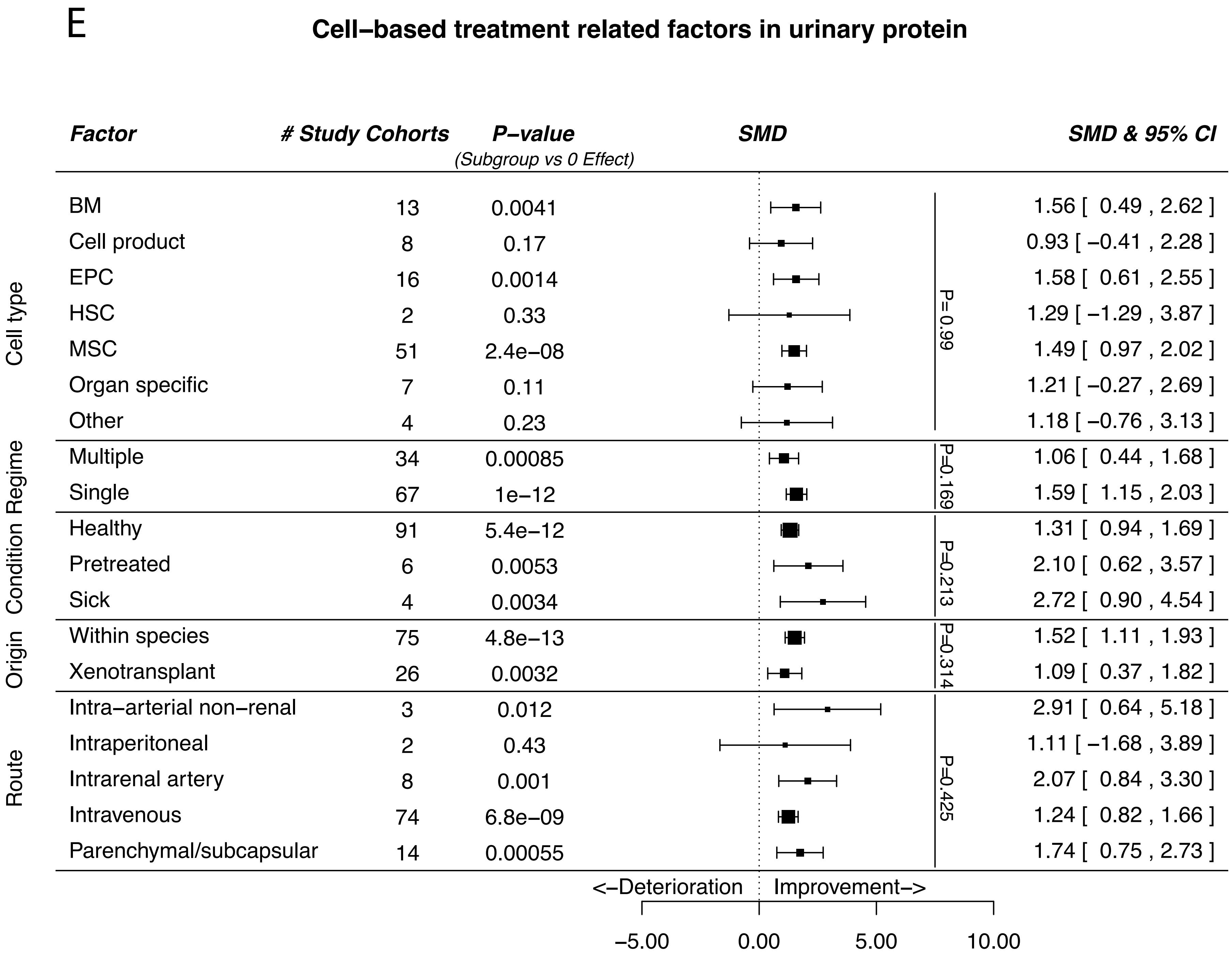

Cell–based treatment related factors in urinary protein
Factor
Study Cohorts
P–value
(Subgroup vs 0 Effect)
SMD
SMD & 95% CI
Cell type
BM 13 0.0041 1.56 [0.49 , 2.62]
Cell product 8 0.17 0.93 [–0.41 , 2.28]
EPC 16 0.0014 1.58 [0.61 , 2.55]
HSC 2 0.33 1.29 [–1.29 , 3.87]
MSC 51 2.4e–08 1.49 [0.97 , 2.02]
Organ specific 7 0.11 1.21 [–0.27 , 2.69]
Other 4 0.23 1.18 [–0.76 , 3.13]
P= 0.99
Regime
Multiple 34 0.00085 1.06 [0.44 , 1.68]
Single 67 1e–12 1.59 [1.15 , 2.03]
P=0.169
Condition
Healthy 91 5.4e–12 1.31 [0.94 , 1.69]
Pretreated 6 0.0053 2.10 [0.62 , 3.57]
Sick 4 0.0034 2.72 [0.90 , 4.54]
P=0.213
Origin
Within species 75 4.8e–13 1.52 [1.11 , 1.93]
Xenotransplant 26 0.0032 1.09 [0.37 , 1.82]
P=0.314
Route
Intra–arterial non–renal 3 0.012 2.91 [0.64 , 5.18]
Intraperitoneal 2 0.43 1.11 [–1.68 , 3.89]
Intrarenal artery 8 0.001 2.07 [0.84 , 3.30]
Intravenous 74 6.8e–09 1.24 [0.82 , 1.66]
Parenchymal/subcapsular 14 0.00055 1.74 [0.75 , 2.73]
P=0.425
<–Deterioration
Improvement–>
–5.00
0.00
5.00
10.00

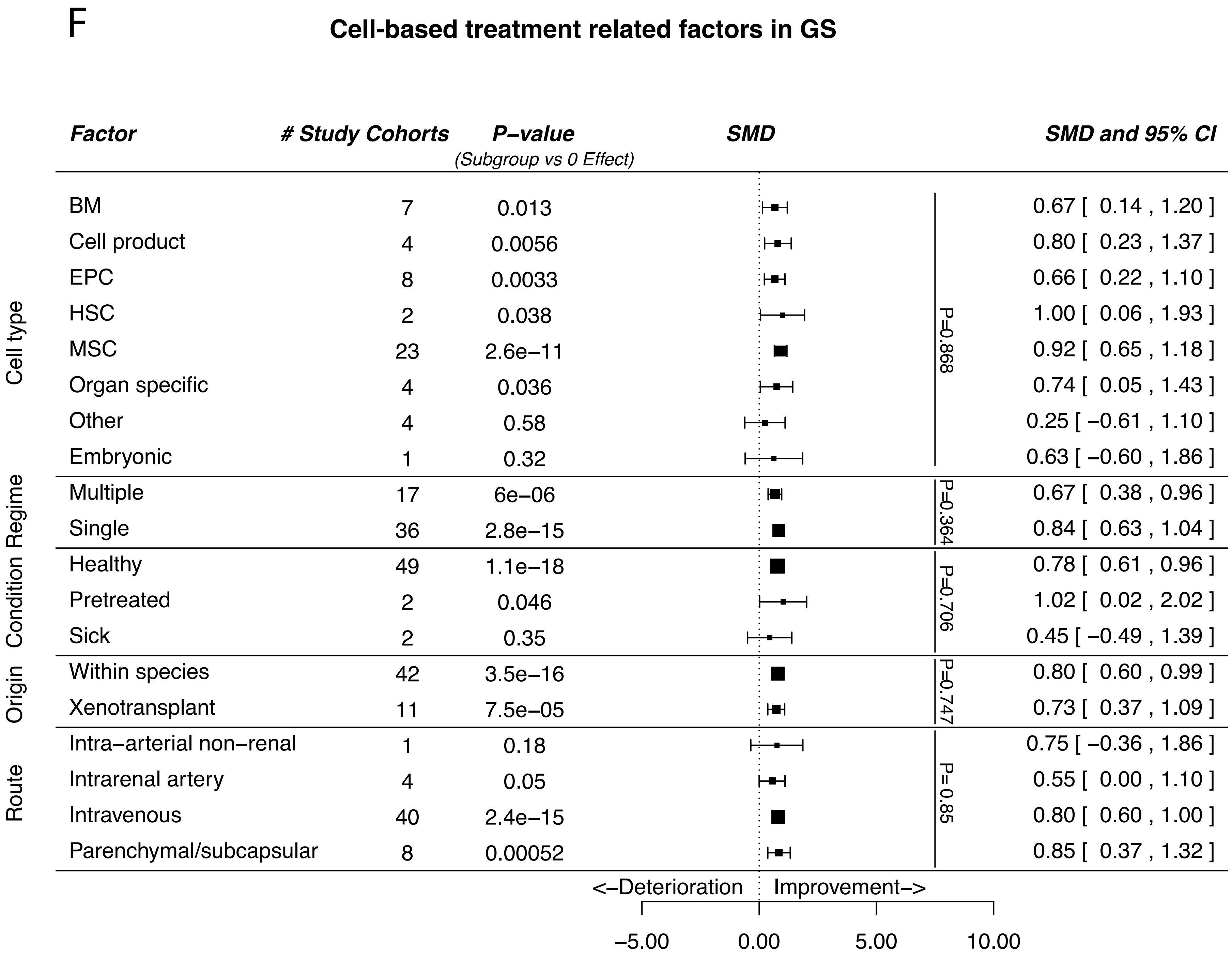
F
Cell-based treatment related factors in GS
Factor
Study Cohorts
P–value
(Subgroup vs 0 Effect)
SMD
SMD and 95% CI
Cell type
BM 7 0.013 0.67 [0.14 , 1.20]
Cell product 4 0.0056 0.80 [0.23 , 1.37]
EPC 8 0.0033 0.66 [0.22 , 1.10]
HSC 2 0.038 1.00 [0.06 , 1.93]
MSC 23 2.6e–11 0.92 [0.65 , 1.18]
Organ specific 4 0.036 0.74 [0.05 , 1.43]
Other 4 0.58 0.25 [–0.61 , 1.10]
Embryonic 1 0.32 0.63 [–0.60 , 1.86]
P=0.868
Regime
Multiple 17 6e–06 0.67 [0.38 , 0.96]
Single 36 2.8e–15 0.84 [0.63 , 1.04]
P=0.364
Condition
Healthy 49 1.1e–18 0.78 [0.61 , 0.96]
Pretreated 2 0.046 1.02 [0.02 , 2.02]
Sick 2 0.35 0.45 [–0.49 , 1.39]
P=0.706
Origin
Within species 42 3.5e–16 0.80 [0.60 , 0.99]
Xenotransplant 11 7.5e–05 0.73 [0.37 , 1.09]
P=0.747
Route
Intra–arterial non–renal 1 0.18 0.75 [–0.36 , 1.86]
Intrarenal artery 4 0.05 0.55 [0.00 , 1.10]
Intravenous 40 2.4e–15 0.80 [0.60 , 1.00]
Parenchymal/subcapsular 8 0.00052 0.85 [0.37 , 1.32]
P= 0.85
<–Deterioration
Improvement–>
–5.00
0.00
5.00
10.00

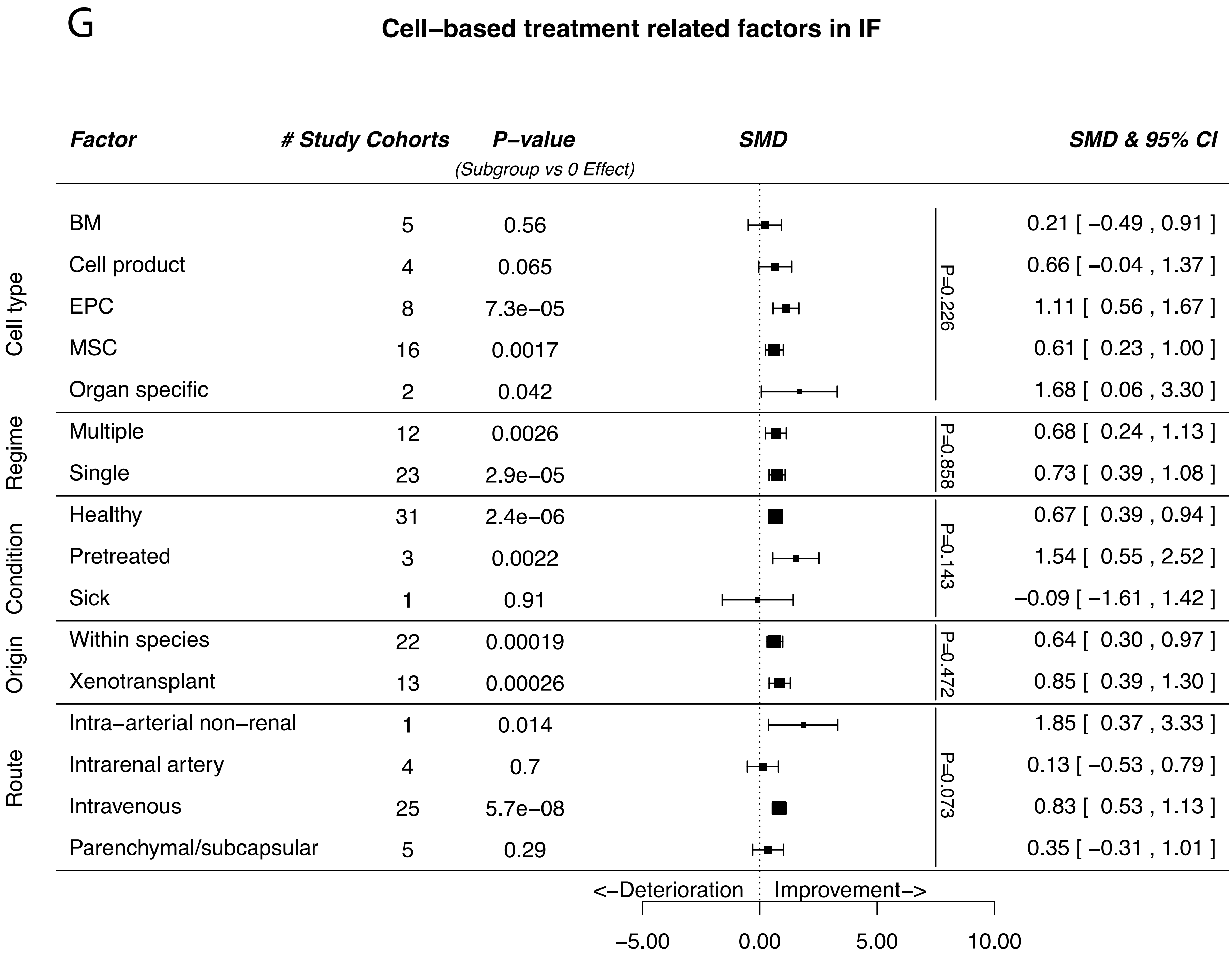
G
Cell–based treatment related factors in IF
Factor
Study Cohorts
P–value
(Subgroup vs 0 Effect)
SMD
SMD & 95% CI
Cell type
BM 5 0.56 0.21 [–0.49 , 0.91]
Cell product 4 0.065 0.66 [–0.04 , 1.37]
EPC 8 7.3e–05 1.11 [0.56 , 1.67]
MSC 16 0.0017 0.61 [0.23 , 1.00]
Organ specific 2 0.042 1.68 [0.06 , 3.30]
P=0.226
Regime
Multiple 12 0.0026 0.68 [0.24 , 1.13]
Single 23 2.9e–05 0.73 [0.39 , 1.08]
P=0.858
Condition
Healthy 31 2.4e–06 0.67 [0.39 , 0.94]
Pretreated 3 0.0022 1.54 [0.55 , 2.52]
Sick 1 0.91 –0.09 [–1.61 , 1.42]
P=0.143
Origin
Within species 22 0.00019 0.64 [0.30 , 0.97]
Xenotransplant 13 0.00026 0.85 [0.39 , 1.30]
P=0.472
Route
Intra–arterial non–renal 1 0.014 1.85 [0.37 , 3.33]
Intrarenal artery 4 0.7 0.13 [–0.53 , 0.79]
Intravenous 25 5.7e–08 0.83 [0.53 , 1.13]
Parenchymal/subcapsular 5 0.29 0.35 [–0.31 , 1.01]
P=0.073
<–Deterioration
Improvement–>
–5.00
0.00
5.00
10.00

Fig. S5. Subgroup analysis of model related factors. Plasma creatinine (A), plasma urea (B), glomerular filtration rate, GFR (C), blood pressure, BP (D), urinary protein (E), glomerulosclerosis, GS (F) and interstitial fibrosis, IF (G). Right side shows improvement by cell-based therapy. Data are presented as SMDs and 95% Cl.

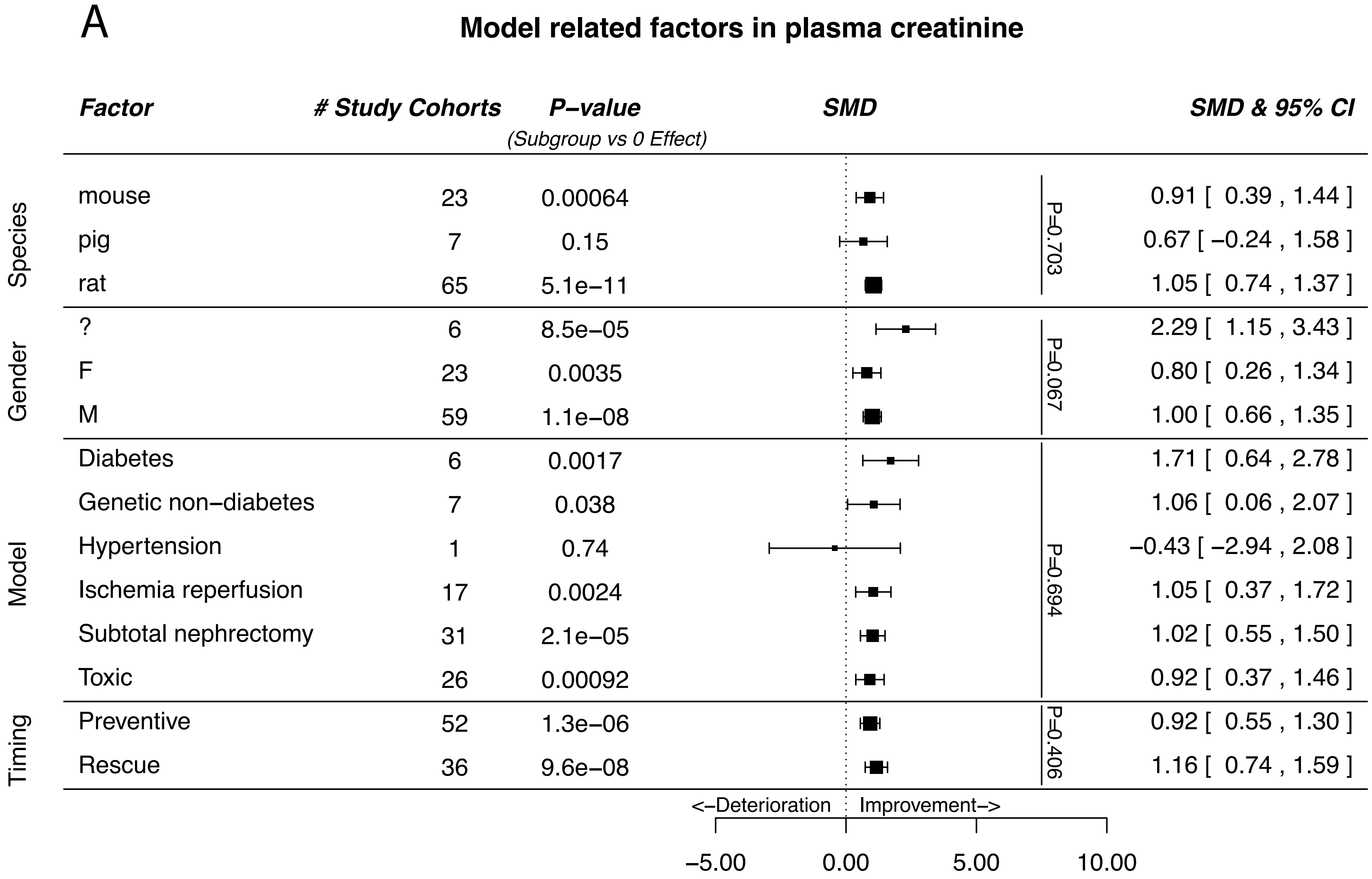

B

Model related factors in plasma urea

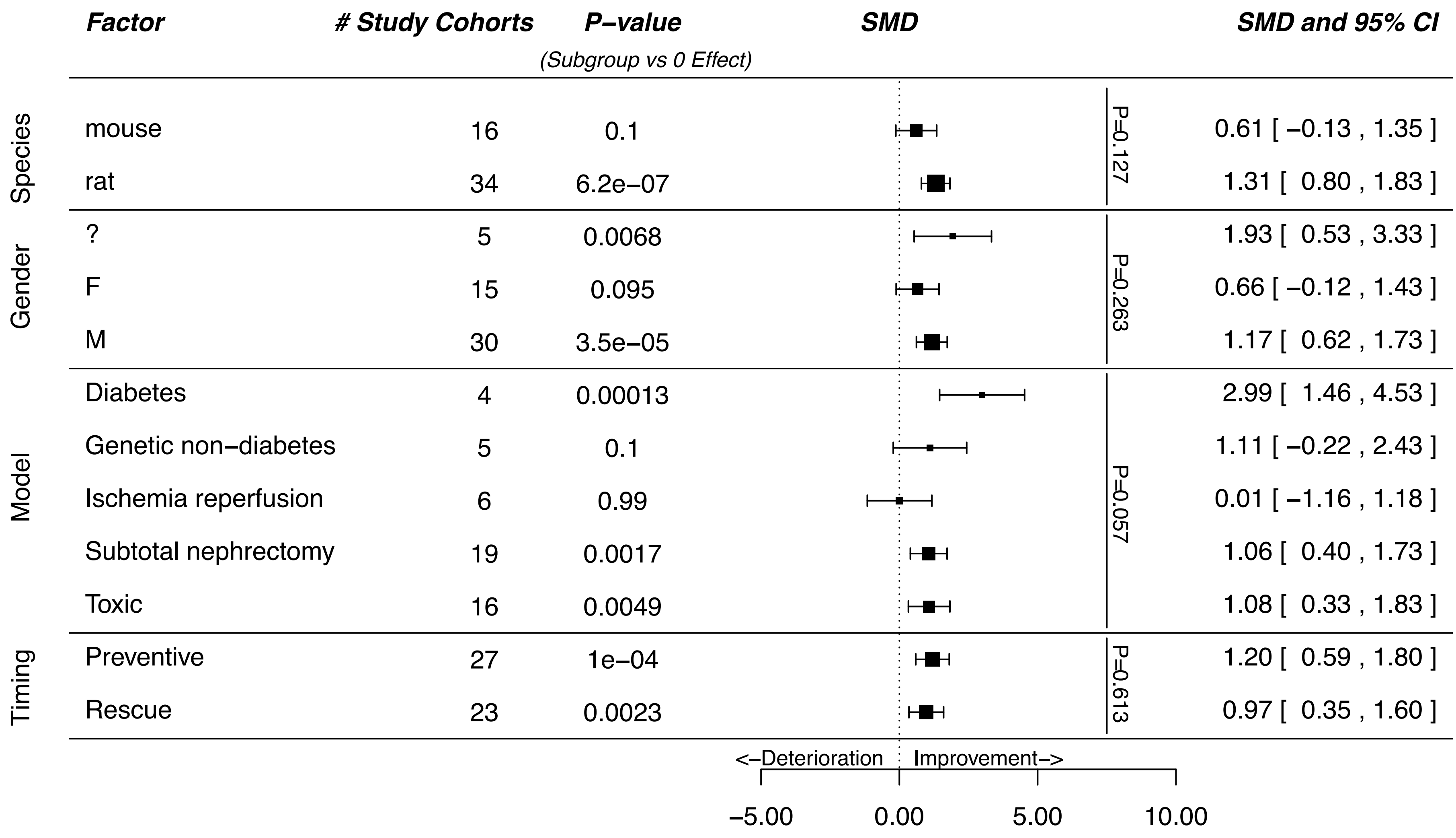

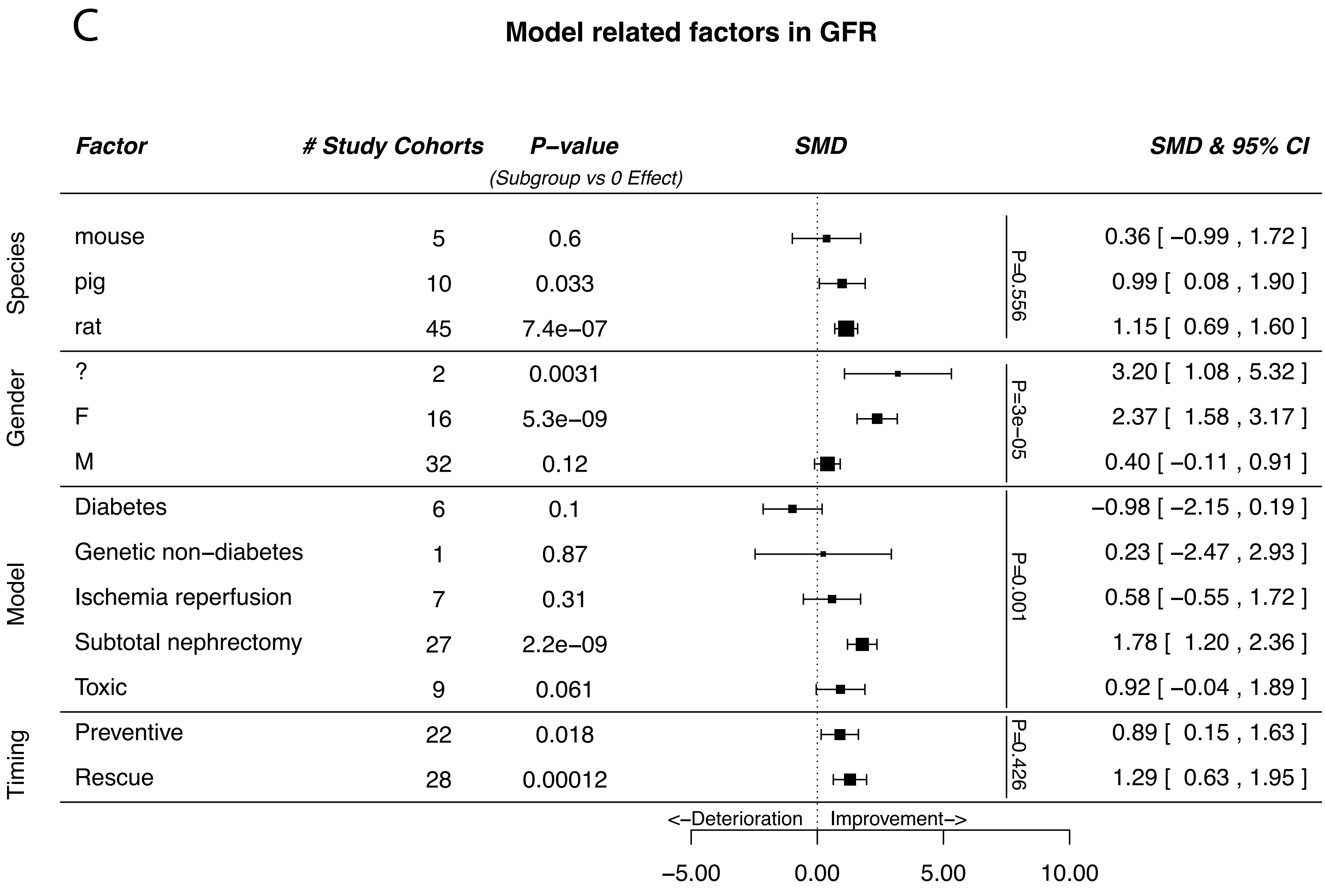
C
Model related factors in GFR
Factor
Study Cohorts
P–value
(Subgroup vs 0 Effect)
SMD
SMD & 95% CI
Species
mouse 5 0.6 0.36 [−0.99 , 1.72]
pig 10 0.033 0.99 [0.08 , 1.90]
rat 45 7.4e−07 1.15 [0.69 , 1.60]
P=0.556
Gender
? 2 0.0031 3.20 [1.08 , 5.32]
F 16 5.3e−09 2.37 [1.58 , 3.17]
M 32 0.12 0.40 [−0.11 , 0.91]
P=3e−05
Model
Diabetes 6 0.1 −0.98 [−2.15 , 0.19]
Genetic non−diabetes 1 0.87 0.23 [−2.47 , 2.93]
Ischemia reperfusion 7 0.31 0.58 [−0.55 , 1.72]
Subtotal nephrectomy 27 2.2e−09 1.78 [1.20 , 2.36]
Toxic 9 0.061 0.92 [−0.04 , 1.89]
P=0.001
Timing
Preventive 22 0.018 0.89 [0.15 , 1.63]
Rescue 28 0.00012 1.29 [0.63 , 1.95]
P=0.426
<−Deterioration
Improvement−>
−5.00
0.00
5.00
10.00

D

Model related factors in BP

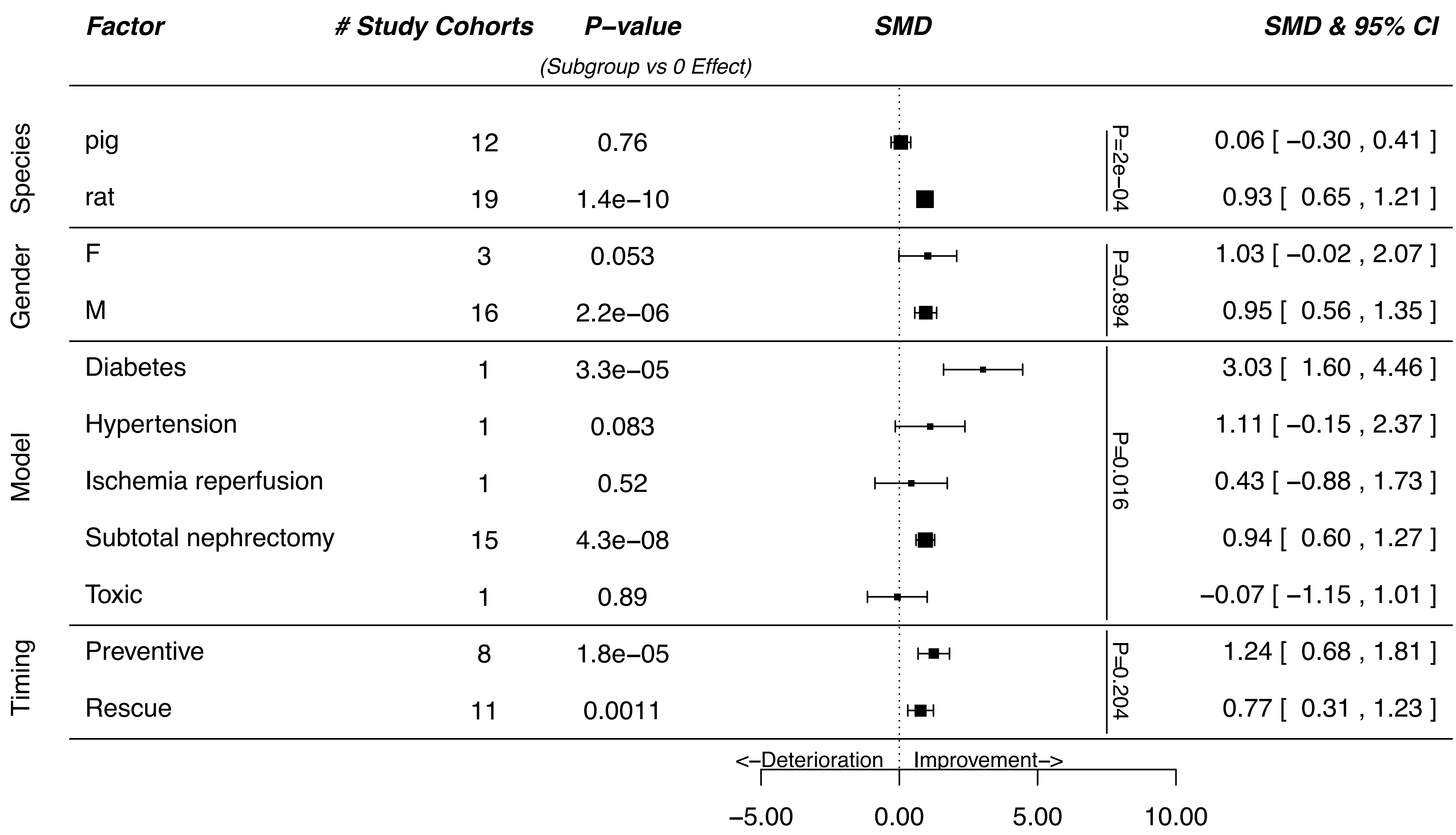

E

Model related factors in urinary protein

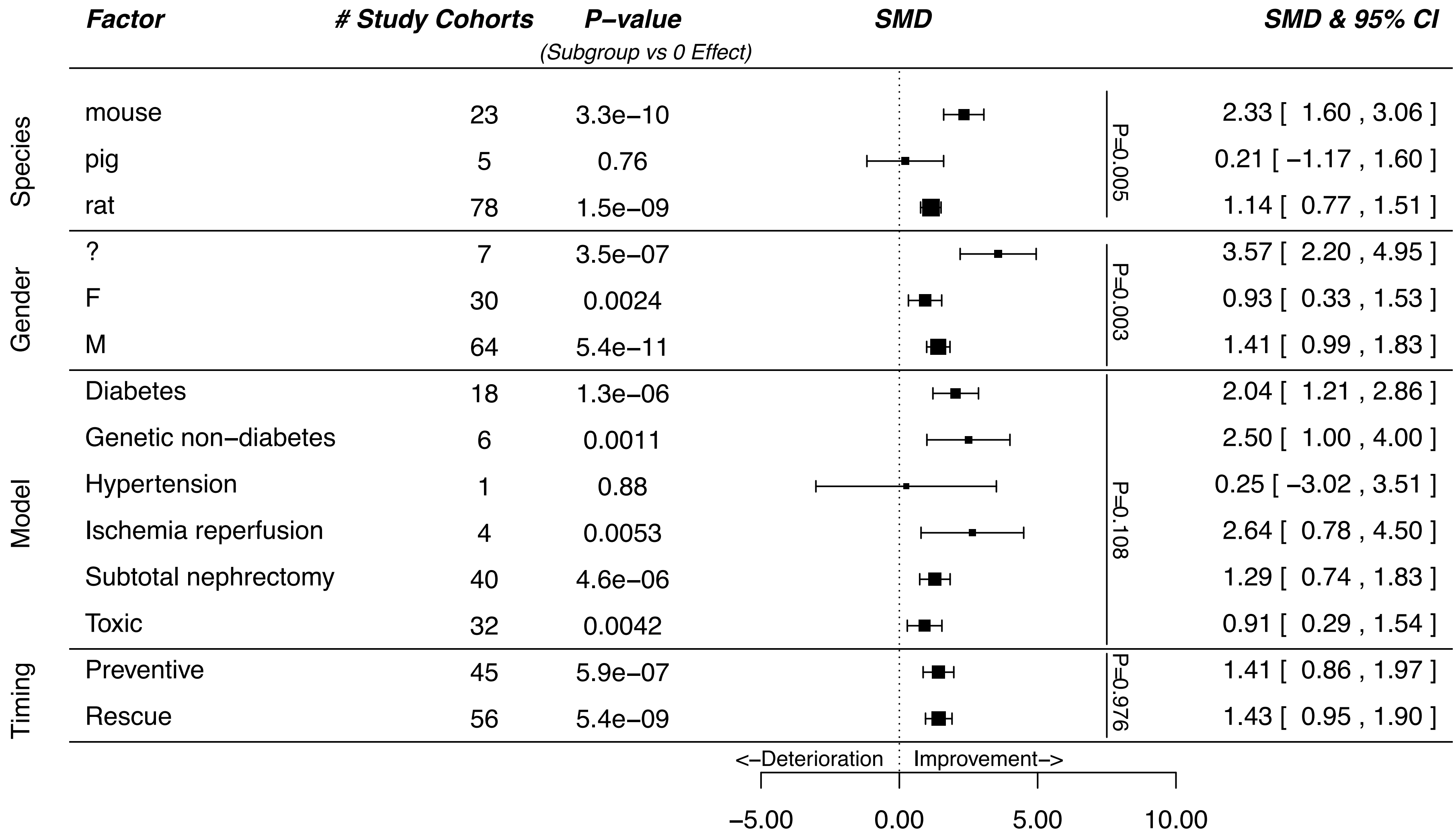

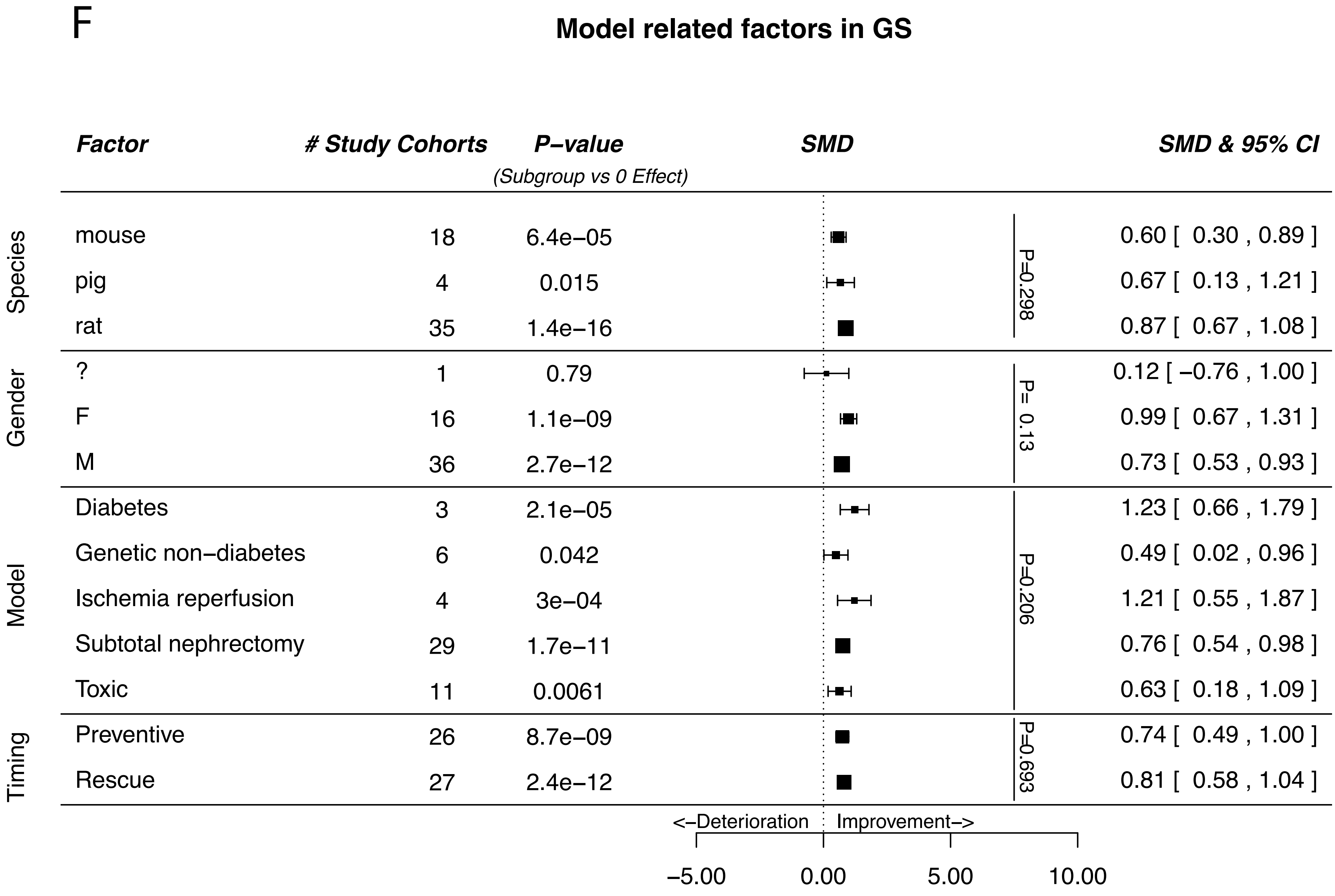
F
Model related factors in GS
Factor
Study Cohorts
P−value
(Subgroup vs 0 Effect)
SMD
SMD & 95% CI
Species
mouse 18 6.4e−05 0.60 [0.30 , 0.89]
pig 4 0.015 0.67 [0.13 , 1.21]
rat 35 1.4e−16 0.87 [0.67 , 1.08]
P=0.298
Gender
? 1 0.79 0.12 [−0.76 , 1.00]
F 16 1.1e−09 0.99 [0.67 , 1.31]
M 36 2.7e−12 0.73 [0.53 , 0.93]
P= 0.13
Model
Diabetes 3 2.1e−05 1.23 [0.66 , 1.79]
Genetic non−diabetes 6 0.042 0.49 [0.02 , 0.96]
Ischemia reperfusion 4 3e−04 1.21 [0.55 , 1.87]
Subtotal nephrectomy 29 1.7e−11 0.76 [0.54 , 0.98]
Toxic 11 0.0061 0.63 [0.18 , 1.09]
P=0.206
Timing
Preventive 26 8.7e−09 0.74 [0.49 , 1.00]
Rescue 27 2.4e−12 0.81 [0.58 , 1.04]
P=0.693
<−Deterioration
Improvement−>
−5.00
0.00
5.00
10.00

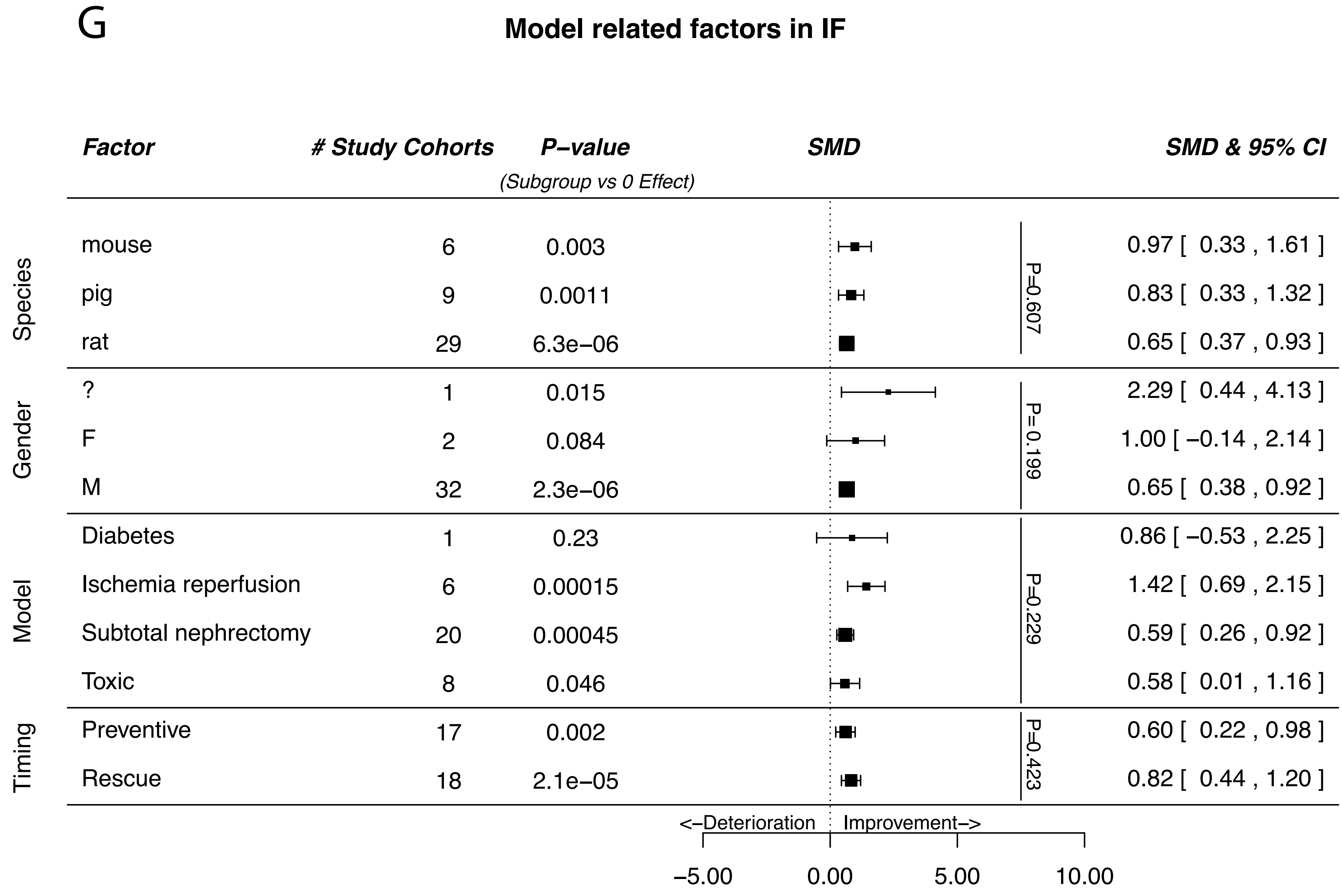
G
Model related factors in IF
Factor
Study Cohorts
P–value
(Subgroup vs 0 Effect)
SMD
SMD & 95% CI
Species
mouse 6 0.003 0.97 [0.33 , 1.61]
pig 9 0.0011 0.83 [0.33 , 1.32]
rat 29 6.3e–06 0.65 [0.37 , 0.93]
P=0.607
Gender
? 1 0.015 2.29 [0.44 , 4.13]
F 2 0.084 1.00 [–0.14 , 2.14]
M 32 2.3e–06 0.65 [0.38 , 0.92]
P= 0.199
Model
Diabetes 1 0.23 0.86 [–0.53 , 2.25]
Ischemia reperfusion 6 0.00015 1.42 [0.69 , 2.15]
Subtotal nephrectomy 20 0.00045 0.59 [0.26 , 0.92]
Toxic 8 0.046 0.58 [0.01 , 1.16]
P=0.229
Timing
Preventive 17 0.002 0.60 [0.22 , 0.98]
Rescue 18 2.1e–05 0.82 [0.44 , 1.20]
P=0.423
<–Deterioration
Improvement–>
–5.00
0.00
5.00
10.00

Fig. S6. Quality assessment score.

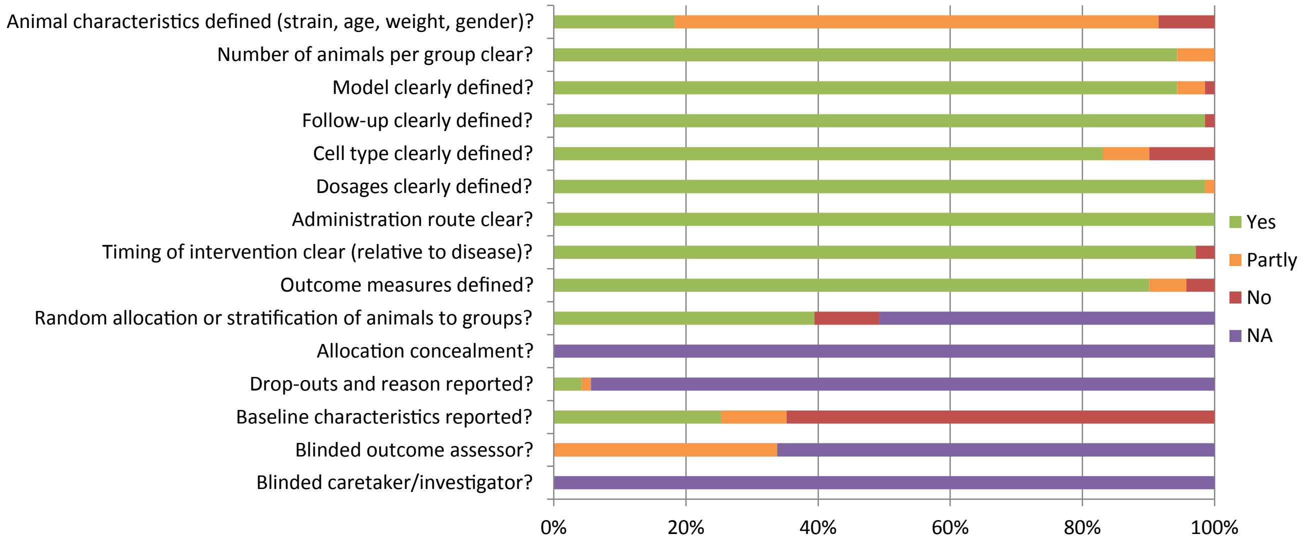

Fig. S7. Funnel plots for plasma creatinine (A), plasma urea (B), GFR (C), BP (D), urinary protein (E), GS (F) and IF (G).

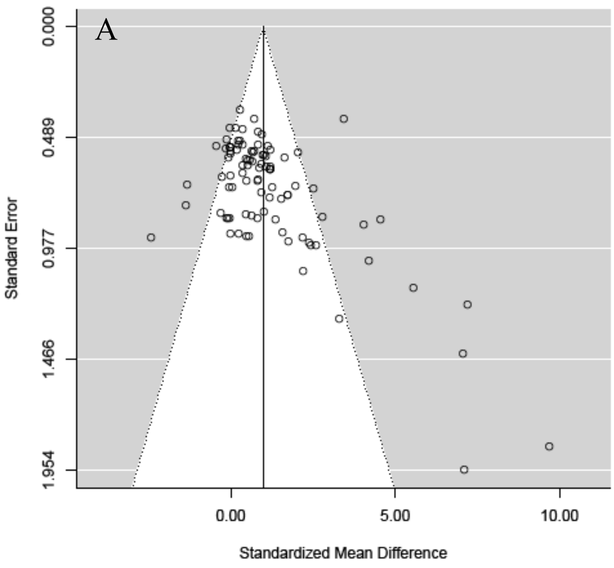

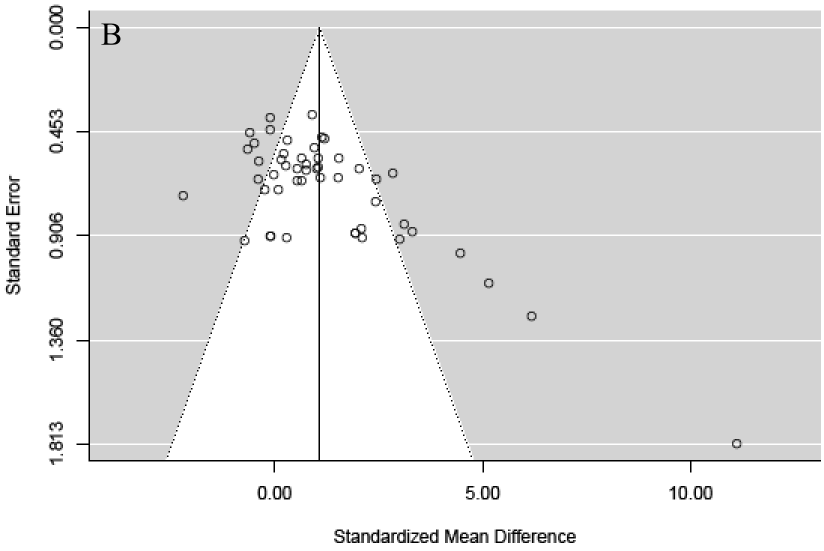

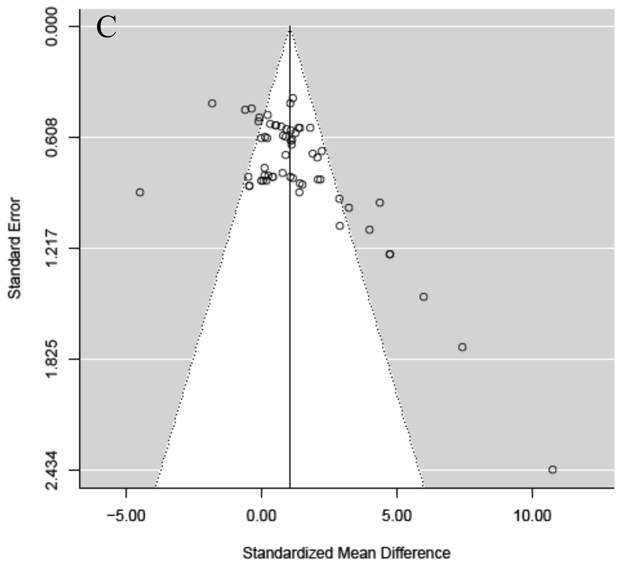
C
Standard Error
0.000
0.608
1.217
1.825
2.434
-5.00
0.00
5.00
10.00
Standardized Mean Difference

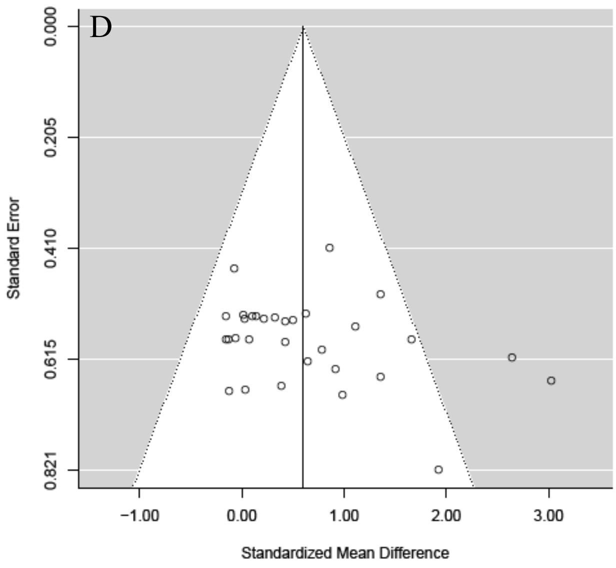
D
Standard Error
0.000
0.205
0.410
0.615
0.821
-1.00
0.00
1.00
2.00
3.00
Standardized Mean Difference

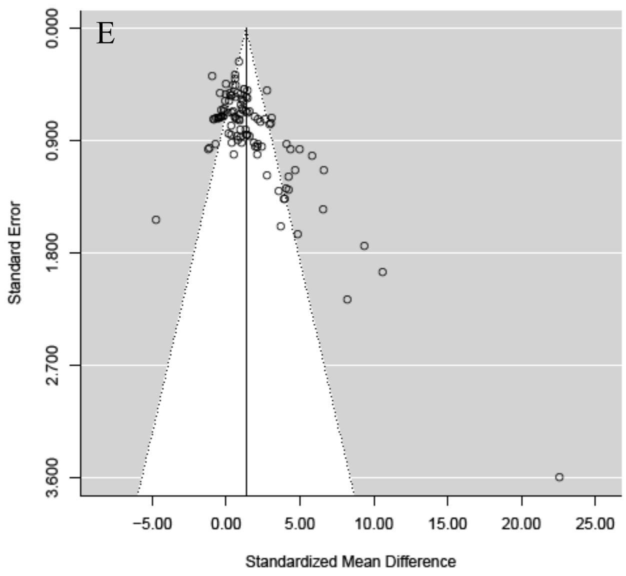
E
Standard Error
0.000
0.900
1.800
2.700
3.600
-5.00
0.00
5.00
10.00
15.00
20.00
25.00
Standardized Mean Difference

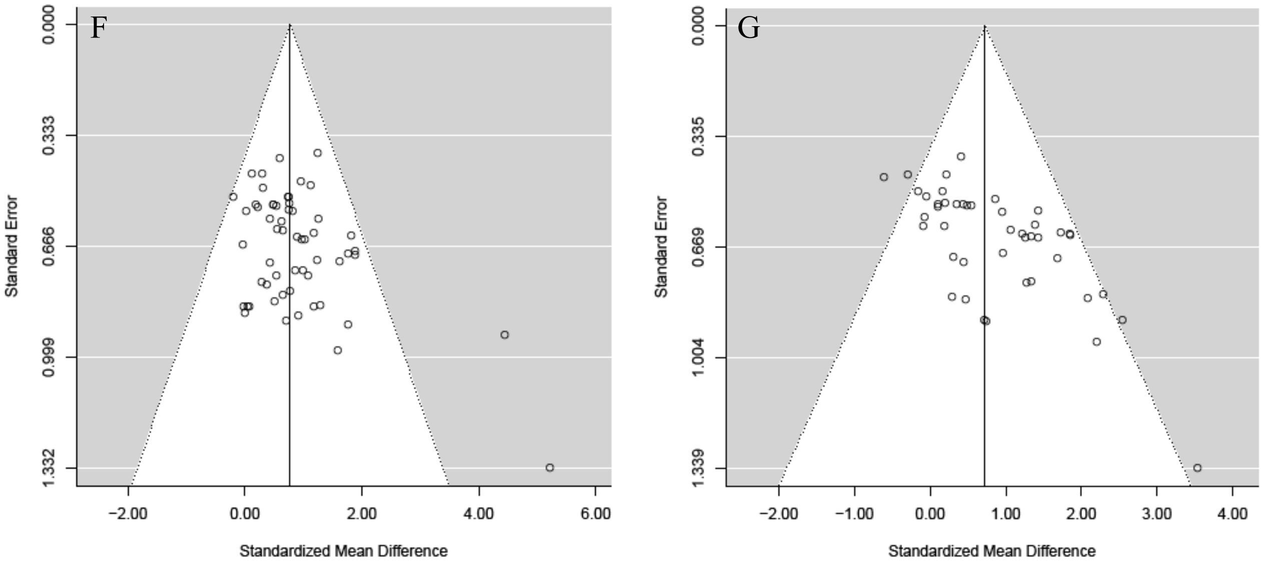

F
G
Standard Error
Standardized Mean Difference
0.000
0.333
0.666
0.999
1.332
-2.00
0.00
2.00
4.00
6.00
0.335
0.669
1.004
1.339
-1.00
1.00
3.00

Fig. S8. Imputed missing studies (open symbols) for plasma creatinine (A), urinary protein (B) and GFR (C).

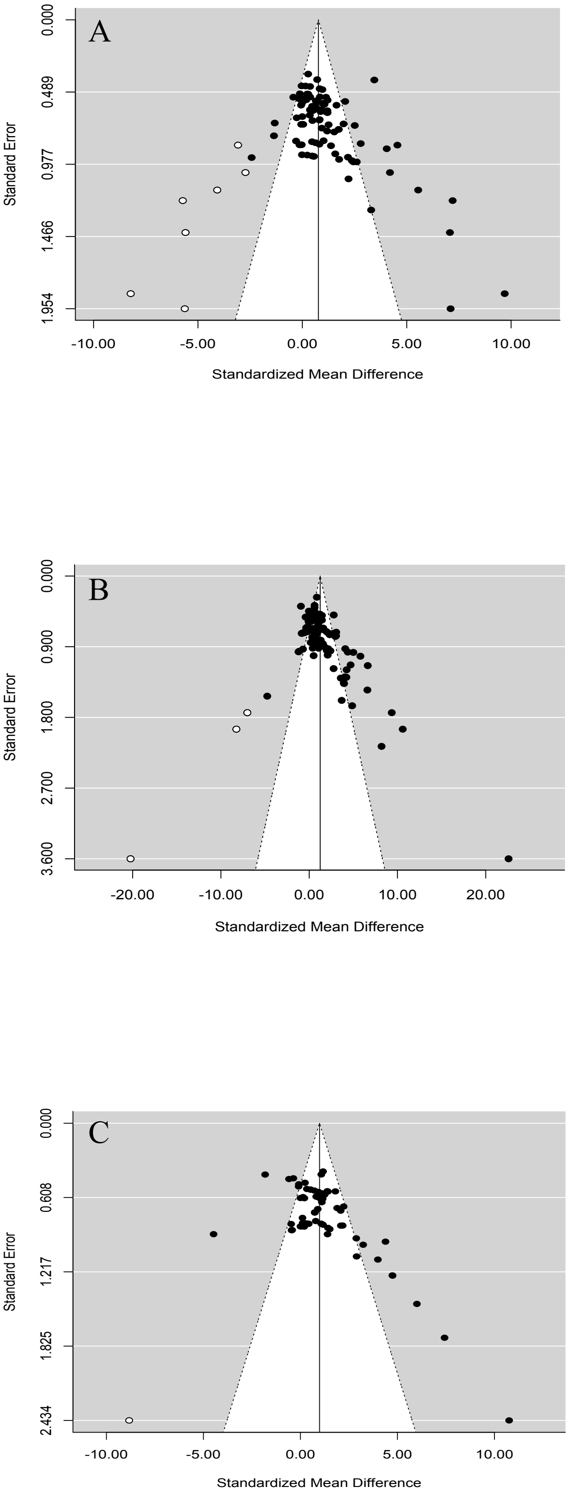

Supplement: Supplementary Material [file supp_8.3.281_DMM017699.pdf]
